# Computational prediction of microRNAs in marine bacteria of the genus *Thalassospira*

**DOI:** 10.1371/journal.pone.0212996

**Published:** 2019-03-12

**Authors:** Thi Hoang Yen Dang, Sonika Tyagi, Glenn D’Cunha, Mrinal Bhave, Russell Crawford, Elena P. Ivanova

**Affiliations:** 1 Swinburne University of Technology, Hawthorn, Australia; 2 Australian Genome Research Facility Ltd, The Walter and Eliza Hall Institute, 1G Royal Pde, Parkville, Melbourne, Victoria, Australia; 3 School of Biological Sciences, Monash University, Clayton, Victoria; 4 RMIT University, Melbourne, Australia; 5 Pacific Institute of Bioorganic Chemistry, Far-Eastern Branch of the Russian Academy of Sciences, Vladivostok, Russian Federation; Massey University, NEW ZEALAND

## Abstract

MicroRNAs (miRNAs) are key players in regulation of gene expression at post-transcription level in eukaryotic cells. MiRNAs have been intensively studied in plants, animals and viruses. The investigations of bacterial miRNAs have gained less attention, except for the recent studies on miRNAs derived from *Streptococcus mutans* ATCC 25175 and *Escherichia coli* DH10B. In this study, high-throughput sequencing approach was employed to investigate the miRNA population in bacteria of the genus *Thalassospira* using both the miRDeep2 and CID-miRNA methods. A total of 984 putative miRNAs were identified in 9 species of the genus *Thalassospira* using both miRDeep and CID-miRNA analyses. Fifty seven conserved putative miRNAs were found in different species of the genus *Thalassospira*, and up to 6 miRNAs were found to be present at different locations in the *T*. *alkalitolerans* JCM 18968^T^, *T*. *lucentensis* QMT2^T^ and *T*. *xianhensis* P-4^T^. None of the putative miRNAs was found to share sequence to the reported miRNAs in *E*. *coli* DH10B and *S*. *mutans* ATCC 25175. The findings provide a comprehensive list of computationally identified miRNAs in 9 bacterial species of the genus *Thalassospira* and addressed the existing knowledge gap on the presence of miRNAs in the *Thalassospira* genomes.

## Introduction

MicroRNA (miRNA) is a class of small, non-coding RNA molecules containing 19–22 nucleotides. Since the discovery of miRNA in *Caenorhabditis elegans* [1], a large number of predicted miRNA molecules has been reported in animals, plants and viruses as key players in regulation of gene expression networks [2–5]. In bacteria, the small non-coding RNAs (sRNAs) have been demonstrated to have a similar function to eukaryotic miRNA, in modulating the target mRNAs in various ways at a post-transcriptional level [6]. A number of sRNAs have been identified in bacteria, some of which were identified in marine bacteria such as *Vibrio* spp. and *Synechococcus* spp. that are functional analogues to plant miRNAs in response to environmental changes [7, 8]. The investigations of bacterial miRNAs, however, have gained little attention, except for the recent studies on miRNAs derived from *Streptococcus mutans* ATCC 25175 and *Escherichia coli* DH10B [9, 10].

The genus *Thalassospira* includes Gram-negative, aerobic and halophilic bacteria dwelling in a marine environment [11]. Bacteria of this genus are involved in the biodegradation of a variety of hydrocarbons [12–14]. For example, *T*. *tepidiphila* 1-1B^T^ and *T*. *povalilytica Zumi* 95^T^ have the ability to degrade polycyclic aromatic hydrocarbons and polyvinyl alcohol [12, 13]; and *T*. *australica* NP 3b2^T^ is able to utilise poly (ethylene) terephthalate (PET) plastic as a carbon source [14]. Currently the genus *Thalassospira* is comprised of 10 validly named species, of which the whole genome sequences of four species (*T*. *australica* NP 3b2^T^, *T*. *lucentensis* QMT2^T^, *T*. *profundimaris* WP0211^T^ and *T*. *xiamenensis* M-5^T^) have been assembled and deposited in public databases [15–17]. The presence of miRNAs in bacteria of this genus has, however, not been demonstrated. Here, the investigation of miRNA populations was carried out using 9 validly named *Thalassospira* species, i.e., *T*. *alkalitolerans* JCM 18968^T^, *T*. *lucentensis* QMT2^T^, *T*. *mesophila* JCM 18969^T^, *T*. *povalilytica* Zumi 95^T^, *T*. *profundimaris* WP0211^T^, *T*. *tepidiphila* 1-1B^T^, *T*. *xiamenensis* M-5^T^, *T*. *xianhensis* P-4^T^ and *T*. *australica* NP 3b2^T^. Since *Thalassospira lohafexi* 139Z-12^T^ was not validly published until 2015 [11], this species was not included in this study. A deep sequencing approach has been used to identify miRNAs in bacteria in other studies [10]. Thus, the aim of this work was to identify the potential miRNAs in bacteria of the genus *Thalassospira* using computational approaches from small RNA sequence dataset generated by high-throughput sequencing technology.

## Materials and methods

### Bacterial strains and growth conditions

Nine type strains of *Thalassospira* species were obtained from various culture collections and used in this study. *T*. *australica* JCM 31222^T^, *T*. *alkalitolerans* JCM 18968^T^, *T*. *mesophila* JCM 18969^T^, *T*. *povalilytica* JCM 18746^T^, *T*. *tepidiphila* JCM 14578^T^ and *T*. *xianhensis* JCM 14850^T^ were obtained from RIKEN BRC-Japan Collection of Microorganisms (JCM) and *T*. *lucentensis* DSM 14000^T^, *T*. *profundimaris* DSM 17430^T^ and *T*. *xiamenensis* DSM 17429^T^ were obtained from German Collection of Microorganisms and Cell Cultures (DSMZ). Bacteria were aerobically grown at their stationary phase in marine broth 2216 (BD, U.S.A.) at 22 ^o^C for 48 h [18, 19]. Bacterial cells were collected by centrifugation and used for RNA extraction or stored at -80°C in marine broth 2216 (BD, USA) supplemented with 20% (v/v) glycerol.

### RNA isolation and construction of small RNA libraries

Total RNAs were extracted from bacterial cells of *Thalassospira* species using TRIsure reagent (Bioline, Australia) according to the manufacturer’s instructions. The RNA concentration and purity were determined by measuring the absorbance at 260 nm and A260/A280 ratio using Nanodrop (ThermoFisher, Australia). The integrity of extracted RNAs was examined by a Bioanalyzer (Agilent Technology, USA). A RIN value ≥ 5 was applied for selection of good RNA quality with RIN (RNA integrity number) generated for each sample based on the ratio of ribosomal bands and the presence or absence of degradation products on the electrophoretic image. Small RNA-Seq libraries were prepared using the TruSeq Small RNA library preparation kit for Illumina (New England Biolabs, USA). Briefly, rRNA was depleted with Ribo Zero. RNAs were fragmented by heat and divalent cations and the 1^st^ strand cDNA was synthesised with SuperScript II Reverse Transcriptase (Invitrogen). The DNA fragments were 3’adenylated, ligated with 3’ and 5’ adapters and amplified via PCR (13 cycles). The amplified products were loaded on 6% polyacrylamide for small RNA (18–35 nucleotides) selection. The amplicons of small RNA were purified and sequenced using Illumina Hiseq2500 sequencing platform with 50 bp single end chemistry. The raw sequencing data generated from this study was submitted to the NCBI Sequence Read Archive (http://www.ncbi.nlm.nih.gov/sra) under accession number PRJNA505357.

### Bioinformatics analysis of bacterial miRNAs

The raw reads (18–35 nucleotides) generated from the Illumina sequencing were first filtered by removing any contaminations including rDNA, Illumina small RNA adapter sequences or low-quality reads with < 18 nt length using Cutadapt [20]. The remaining reads after filtering were then aligned against the *Thalassospira* genome sequences available in the NCBI database using Bowtie (version 1.1.2) with parameters setting (-n 0 -e 80 -l 18 -a -m 5—best–strata) [21]. The whole genome sequences used as references for these 9 *Thalassospira* species were listed in **S1 Table**. The mapped sequences to genomes were further processed to remove redundancy and normalised expression was computed as CPM (Reads counts per Million) [22]. After normalisation, these mapped sequences were used to predict miRNAs. Two different miRNA prediction algorithms, miRDeep2 and CID-miRNA were used to detect miRNA in this study. The miRDeep2 software (version 2.0.0.7, http://www.mdc-berlin.de/rajewsky/miRDeep) was utilised to predict miRNAs based on an investigation of the secondary structure of the miRNA precursor sequences and integration of miRNA precursors with Dicer, providing the precursors and mature miRNA sequences [23]. CID-miRNA [24] is a method to identify miRNA precursors based on secondary structure filter and an algorithm of stochastic context free grammar (SCFG) [24]. This method only predicts the miRNA precursors. Therefore, *MatureBayes* (http://mirna.imbb.forth.gr/MatureBayes.html) was employed to identify mature miRNAs based on the sequence and structure of the miRNA precursors provided by CID-miRNA [25]. The small sequences mapped to the genome were processed through miRDeep2 ver2.0.0.7 as well as CID-miRNA and *MatureBayes* for identifying mature miRNAs with default parameters. The detected mature miRNAs were further investigated by searching any shared miRNA sequences generated in both methods using BLAST algorithm (version 4) with default setting. The potential precursor sequences predicted by both methods were also BLAST-searched to find any shared precursor sequences. Any shared miRNA sequences were then confirmed using ClustalW2 (https://www.ebi.ac.uk/Tools/msa/clustalw2/) with default setting. In order to find any conserved miRNA across the genus, the predicted mature miRNAs were aligned to those reported in *Escherichia coli* DH10B and *Streptococcus mutans* ATCC 25175 [9, 10]. The candidates were also aligned to miRNAs predicted within the genus *Thalassospira* to identify any conserved miRNAs across all bacteria within the genus.

### Phylogenetic analysis and genome comparison

The 16S rRNA gene sequences of validly described *Thalassospira* species were collected from NCBI (https://www.ncbi.nlm.nih.gov/) and compared using the ClustalW2 (https://www.ebi.ac.uk/Tools/msa/clustalw2/) with default setting. Evolutionary phylogenetic trees were then generated using the neighbour-joining (NJ) algorithm [26]. Genetic distances for the NJ tree were calculated using Kimura’s two-parameter model [27], with the robustness of 1,000 replications, using MEGA 7 software [28].

## Results and discussion

### Generation of small RNA libraries and data evaluation

Small RNA-seq libraries were constructed for nine *Thalassospira* species, including *T*. *alkalitolerans* JCM 18968^T^, *T*. *lucentensis* QMT2^T^, *T*. *mesophila* JCM 18969^T^, *T*. *povalilytica* Zumi 95^T^, *T*. *profundimaris* WP0211^T^, *T*. *tepidiphila* 1-1B^T^, *T*. *xiamenensis* M-5^T^, *T*. *xianhensis* P-4^T^ and *T*. *australica* NP 3b2^T^. Next-generation small RNA-seq was used for detection of the presence of small RNAs. The results yielded 242,204,737 sequence reads of 18–33 nucleotides (nt) length, ranging from 22.99 to 31.43 million reads for 9 libraries (**Table 1**). The reads were curated for any contamination or artefacts such as rDNA or Illumina small RNA adaptor sequences using Cutadapt [20]. The cleaned reads were then aligned to the *Thalassospira* genome sequences available in the NCBI database using Bowtie software [21].

**Table 1:** Bacterial small RNA-Seq data yield from Hiseq2500 sequencing.

Samples	*T*. *australica* NP 3b2^T^	*T*. *alkalitolerans* JCM 18968^T^	*T*. *lucentensis* QMT2^T^	*T*. *mesophila* JCM 18969^T^	*T*. *povalilytica* Zumi 95^T^	*T*. *profundimaris* WP0211^T^	*T*. *tepidiphila* 1-1B^T^	*T*. *xiamenensis* M-5^T^	*T*. *xianhensis* P-4^T^
**Original number of reads**^a^	22,991,595	31,431,409	28,737,289	27,213,115	28,225,762	25,272,618	25,641,708	28,144,650	24,546,591
**Reads after filter and collapse**^b^	3,177,403	5,068,260	4,606,730	2,954,668	3,761,719	5,327,201	4,368,445	1,712,391	3,300,999
**Reads aligning to reference genome**^c^	2,285,671	160,990	2,511,917	102,497	95,972	2,046,567	1,230,299	1,143,173	631,902
**Aligned reads, %**	71.94	3.18	54.53	3.47	2.55	38.42	28.16	66.76	19.14

^a^: total reads obtained from different bacterial species

^b^: reads obtained after filtering

^c^: reads were aligned to the *Thalassopsira* genome sequences using the Bowtie algorithm. The reads obtained from this experiment were set from 18–33 nt using an Illumina genome analyser with a low error rate (0.005 error per base).

Since the whole genome sequences of *T*. *australica* NP 3b2^T^, *T*. *lucentensis* QMT2^T^, *T*. *profundimaris* WP0211^T^ and *T*. *xiamenensis* M-5^T^ were available in the NCBI database, the small RNA sequence reads were aligned to these genome sequences. The remaining 5 *Thalassospira* species that did not have the whole genome sequence were aligned to these references based on their phylogenetically closest species [14] (**S1 Table, S1 Fig**). By aligning 34.23 million collapsed reads from nine species of the genus *Thalassospira* to the genome sequences available in NCBI, it was found that a proportion of cleaned reads was mappable to the reference genomes, ranging from 38.42% to 71.94% for 4 species with available genome sequences and from 2.55% to 28.16% for the remaining 5 species without whole genome sequenced (**Table 1**). The aligned reads were further analysed for miRNA identification.

### Identification of miRNAs using the mirDeep2 method

**Fig 1:**
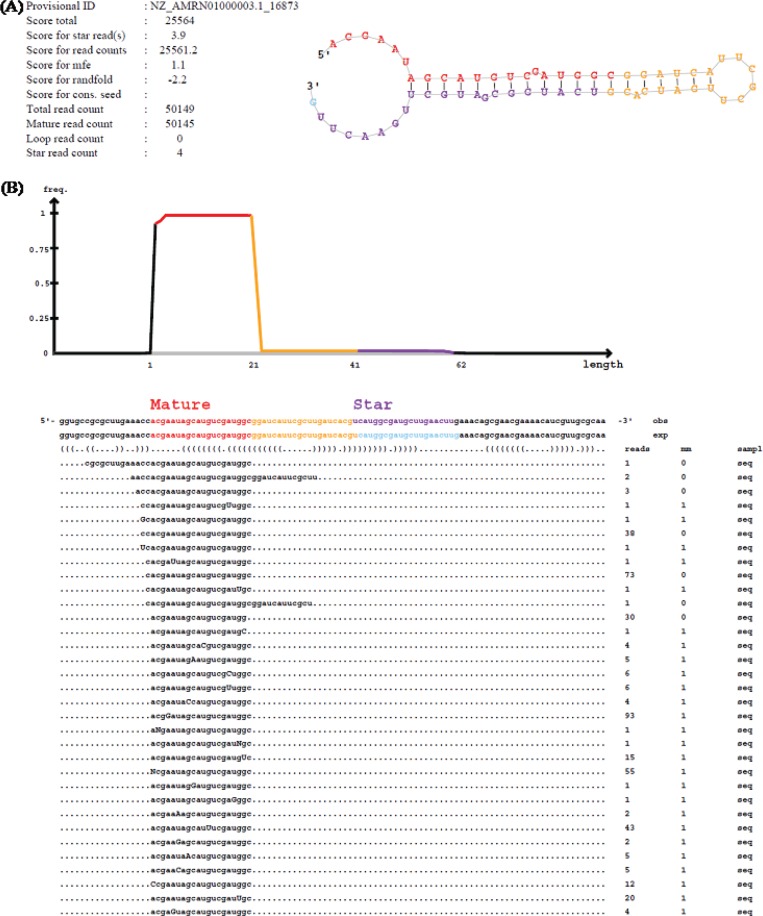
An example of miRDeep output for T.prof_5p_16873. (A) Predicted secondary structure of miRNA precursor with the mature, star and loop sequences (highlighted in red, yellow and purple, respectively). The scores associated to the miRNA, the read counts for mature, star and loop sequences and the total read count are also given. (B) Sequences of the predicted miRNA precursors mapped to the mature, star and loop sequences on the genome (obs line) and the experimental sequence reported in miRBase (exp line). The frequency (reads column) and mismatches of the read with the genomic sequence (mm column) are also given with the mismatches shown in capital letters.

Identification of bacterial miRNAs is in early stages in comparison to that in animals and plants. Therefore, no specific method has been developed for investigation of miRNAs in bacteria. miRDeep2 software (http://www.mdc-berlin.de/rajewsky/miRDeep) was first presented as an algorithm to predict miRNA in human and animals [23]. This software was later used to successfully identify miRNAs in plant [29]. Based on the applicability in different organisms, miRDeep2 software was, therefore, employed to detect putative bacterial miRNAs from the millions of short sequences generated from the dataset. The miRDeep2 software was designed to detect putative miRNAs based on an investigation of the secondary structure of the miRNA precursor sequences. The potential miRNA precursors were then integrated with a model for miRNA precursor processing using Dicer, releasing the mature miRNA sequences, star sequences (also called miRNA5p and miRNA3p sequences) and the loop [23]. By using miRDeep2, 86 potential miRNA precursor sequences could be identified from the dataset. The mature miRNA, star sequence and the loop were also identified within each precursor (**Fig 1**).

The identified 86 putative mature miRNAs were located in either the 5’ arm or the 3’ arm of the precursors, with 11 sequences from *T*. *australica* NP 3b2^T^, 11 from *T*. *lucentensis* QMT2^T^, 14 from *T*. *profundimaris* WP0211^T^, 6 from *T*. *xiamenensis* M-5^T^, 11 from *T*. *alkalitolerans* JCM 18968^T^, 5 from *T*. *mesophila* JCM 18969^T^, 4 from *T*. *povalilytica* Zumi 95^T^, 9 from *T*. *tepidiphila* 1-1B^T^ and 15 from *T*. *xianhensis* P-4^T^ (**Table 2**) being obtained.

**Table 2:** miRNAs of bacteria of the genus *Thalassospira* identified using miRDeep2.

miRNA name*	Location	Sequence (5’-3’)	Length (nucleotides)	Number of reads
***T*. *australica* NP 3b2**^**T**^				
T.aust_3p_10018	JRJE01000008.1_scaffold_30_10018	TAACGTCTGTCCTTCGGATT	20	2252
T.aust_5p_26441	JRJE01000032.1_scaffold_0_26441	CTTGGCAGGCTGGGCGCTCC	20	845
T.aust_3p_11556	JRJE01000009.1_scaffold_3_11556	TTTTGACTGGATCGGCAACCGTGAT	25	151
T.aust_5p_26002	JRJE01000032.1_scaffold_0_26002	TTTGGCGGGGTCGGGAACC	19	88
T.aust_5p_15617	JRJE01000022.1_scaffold_18_15617	ATCCTCTCCCCGCAACCA	18	2888
T.aust_5p_9704	JRJE01000008.1_scaffold_30_9704	ATTGGCGTCACAGATCAGGGGCAT	24	15
T.aust_3p_23062	JRJE01000031.1_scaffold_1_23062	GAAATCCCTGATCGCGCAG	19	13
T.aust_5p_10770	JRJE01000009.1_scaffold_3_10770	ACAAATCTCGGCAAGGCC	18	229
T.aust_5p_13346	JRJE01000019.1_scaffold_20_13346	CGAACTCTGCACCAAGGC	18	12
T.aust_5p_22895	JRJE01000031.1_scaffold_1_22895	CGAATCTCTCATCACCCACCA	21	4199
T.aust_5p _5988	JRJE01000005.1_scaffold_5_5988	AAACCGGATCCTGCAGCC	18	9
***T*. *alkalitolerans* JCM 18968**^**T**^				
T.alka_5p_5662	ATWN01000006.1_5662	TCACCGGTTGGGAAGGCGCTGA	22	15
T.alka_3p_6962	ATWN01000007.1_6962	CTTCCCGCCCCATGGCCGA	19	14
T.alka_3p_6323	ATWN01000007.1_6323	AATTAATGGGTCCTGACC	18	336
T.alka_5p_4136	ATWN01000004.1_4136	TTTCGGGTGGGCAGCGCC	18	1
T.alka_3p_1438	ATWN01000001.1_1438	CGCATGGGCGGAGCTTTTCGTTAG	24	13
T.alka_3p_6324	ATWN01000007.1_6324	AATTAATGGGTCCTGACC	18	336
T.alka_3p_2121	ATWN01000002.1_2121	ATTGATTGCGGCCATCCG	18	11
T.alka_5p_4462	ATWN01000005.1_4462	CAAGAACCGCCATCTGCATGCC	22	9
T.alka_5p_1889	ATWN01000002.1_1889	ATGCTTTTTGGCCGCATT	18	1
T.alka_3p_75	ATWN01000001.1_75	CGAGGTCGAACATGATGAA	19	25
T.alka_5p_3621	ATWN01000003.1_3621	ATGTTGCCGGTGCGGCGGCGGGC	23	7
***T*. *lucentensis* QMT2**^**T**^				
T.luce_5p_27853	ATWN01000011.1_27853	CCGAGGTCCGGTATCGCCTGACT	23	13160
T.luce_5p_31831	ATWN01000015.1_31831	ATCGTGGCCGCACTGGAGCC	20	907
T.luce_3p_14305	ATWN01000004.1_14305	CGCGCAGGCGGGGATCTCGAGC	22	1860
T.luce_5p_11956	ATWN01000003.1_11956	ATCGCTGCGGGCAATAAAAGACC	23	60
T.luce_5p _2660	ATWN01000001.1_2660	TTACCCGTGAGGTCGGCTGTGCGAT	25	163
T.luce_5p_10500	ATWN01000003.1_10500	ATAATGACGTCCGTTGCGAC	20	922
T.luce_3p_13875	ATWN01000004.1_13875	AAACGGGGTCGGGGGGCTG	19	3395
T.luce_3p_12006	ATWN01000003.1_12006	ACCACAGGTGCGGGCATGGGCATG	24	158
T.luce_5p_14218	ATWN01000004.1_14218	AAAGCCCCGGCGCGATTGTCC	21	158
T.luce_3p_14713	ATWN01000004.1_14713	TTTGCGCGATGGGTCCCTGAT	21	17
T.luce_3p_22524	ATWN01000007.1_22524	TCACAGTCGAGACGCTCTCTCACC	24	50057
***T*. *mesophila* JCM 18969**^**T**^				
T.meso_5p_3464	ATWN01000007.1_3464	ATAAGGAGTAGGCGAATGAGC	21	69
T.meso_3p_3562	ATWN01000007.1_3562	TCACAGTCGAGACGCTCTCTCACC	24	10
T.meso_3p_3026	ATWN01000006.1_3026	CTTGGCGTCGAAGGCATGA	19	2
T.meso_5p_2868	ATWN01000006.1_2868	TTTGGCAAGGCACAGCGCGCAG	22	9
T.meso_3p_3086	ATWN01000006.1_3086	CTGCGCGCTGTGCCTTGCC	19	9
***T*. *povalilytica* Zumi 95**^**T**^				
T.pova_5p_4964	AMRN01000014.1_4964	ATCTTTCGATGGTCGTGGCA	20	251
T.pova_5p_4720	AMRN01000012.1_4720	CCAAGCGCGGTGCGGACCG	19	21
T.pova_5p_661	AMRN01000001.1_661	ATGGGCATCCTGACCGAAGGCACG	24	8
T.pova_5p_390	AMRN01000001.1_390	CTTGAAGACCTGCATCAGCGTTC	23	7
***T*. *profundimaris* WP0211**^**T**^				
T.prof_5p_16873	AMRN01000003.1_16873	ACGAATAGCATGTCGATGGC	20	50145
T.prof_5p_35057	AMRN01000009.1_35057	ATCGCCTGAACGCGCGCCTGACCG	24	1316
T.prof_5p_36012	AMRN01000010.1_36012	GTCCGGTGGTCTGGGCACCATG	22	1679
T.prof_5p_14768	AMRN01000003.1_14768	ATCCTGCCCCCGCAACCA	18	721
T.prof_5p_660	AMRN01000001.1_660	CTATGCAGACACCCCGGAC	19	1702
T.prof_5p_26017	AMRN01000006.1_26017	ATCACGTTGAGCCAAAAGAAAAGC	24	18
T.prof_5p_15266	AMRN01000003.1_15266	ATACAACTGATGTCGCCTGC	20	693
T.prof_3p_2147	AMRN01000001.1_2147	ATCCTCGGAATAGGTATAGGCTTCC	25	57
T.prof_3p_20338	AMRN01000004.1_20338	ATCAATCGCCGGGATCATGATCCC	24	76
T.prof_5p_12543	AMRN01000002.1_12543	ATATACGGCCTGGCATAATC	20	2
T.prof_5p_2228	AMRN01000001.1_2228	TTTGCGGAATGCCACCCGGCAACG	24	10
T.prof_5p_19418	5’ AMRN01000004.1_19418	GTGTTCTTTTGGTCGCGCATGCCG	24	11
T.prof_3p_6436	AMRN01000001.1_6436	AAAAGACCGTCCTGCCACCG	20	6
T.prof_3p_49	AMRN01000001.1_49	CTCCTGAGCCGGGCCAAT	18	9
***T*. *tepidiphila* 1-1B**^**T**^				
T.tepi_5p_8152	AMRN01000002.1_8152	ATAATGACGTCCGTTGCGA	19	90
T.tepi_5p_16607	AMRN01000006.1_16607	TTCAAGTCTGATGCCCGCGCC	21	9
T.tepi_5p_22622	AMRN01000010.1_22622	GTCCGGTGGTCTGGGCACCATG	22	252
T.tepi_5p_2762	AMRN01000001.1_2762	GCAGTGGCTTGGCGGGATCGGGAT	24	148
T.tepi_5p_19992	AMRN01000008.1_19992	GGGCCGAGATCGAAAGCAACACG	23	17
T.tepi_5p_8570	AMRN01000003.1_8570	ATCCTGCCCCCGCAACCA	18	1135
T.tepi_5p_11638	AMRN01000004.1_11638	TTTGTCGTTCTGGGCTGGCA	20	12
T.tepi_5p_21088	AMRN01000009.1_21088	ATTGATATCGCATCGGTTACCGA	23	65
T.tepi_5p_23416	AMRN01000011.1_23416	GTATATTGCCAATTTTGT	18	88
***T*. *xiamenensis* M-5**^**T**^				
T.xiam_3p_12910	CP004388.1_12910	CTTGCCGCCGGTATGCTCGCATC	23	2582
T.xiam_5p_18209	CP004388.1_18209	ATGCAGATCGGTTTGCGCACC	21	47
T.xiam_5p_1950	CP004388.1_1950	ACGGTTTGCGTCGGTCACGCTGGC	24	331
T.xiam_5p_12633	CP004388.1_12633	ATGGACTCCCGCTTTCGC	18	12
T.xiam_5p_12105	CP004388.1_12105	ATTTGCATGCCCGTCTGGC	19	13
T.xiam_3p_3097	CP004388.1_3097	AGCATTCAAGCATCGGCGGGAT	22	13
***T*. *xianhensis* P-4**^**T**^				
T.xian_5p_4477	CP004388.1_4477	TAGGCGGGAGTCCACCGGGC	20	5
T.xian_3p_19710	CP004388.1_19710	GGATCAGCTGGGTAACATC	19	11
T.xian_3p_20546	CP004388.1_20546	CTTGCACCGGGCCGCTTTCGGATG	24	14
T.xian_3p_19849	CP004388.1_19849	ACGCGACCGCGGCAAGGAAA	20	24
T.xian_5p_25207	CP004388.1_25207	AAAGCAGGAAGAATACGAACAGA	23	27
T.xian_5p_31617	CP004388.1_31617	ATGCACCCGGACCGAAACCC	20	558
T.xian_3p_3269	CP004388.1_3269	AAAGCGCGCCCCCTTGCTCCC	21	43
T.xian_5p_9480	CP004388.1_9480	ATTCAGGAATCTGTTCTGACGCAGC	25	26
T.xian_5p_19360	CP004388.1_19360	ATTTTAGTCCGCGTCGCAAC	20	13
T.xian_3p_38616	CP004388.1_38616	CAAACAGCTGAAGGCCTCCC	20	166
T.xian_3p_35228	CP004388.1_35228	ATTCCGATGATCTGGTGATTG	21	11
T.xian_3p_13589	CP004388.1_13589	TTGCCGATCATCGCCCTTGCCCTG	24	32
T.xian_5p_34659	CP004388.1_34659	CTTCAGTTCCTCGACCTT	18	13
T.xian_3p_4479	CP004388.1_4479	CTGACTGGATTCCCGCGT	18	9
T.xian_3p_32022	CP004388.1_32022	ATCATGCCGGGCAGATCA	18	8

*: The identified miRNAs were named as <name of bacteria>_<location of miRNA in the precursor>_<position of miRNA in the genome>.

**Fig 2:**
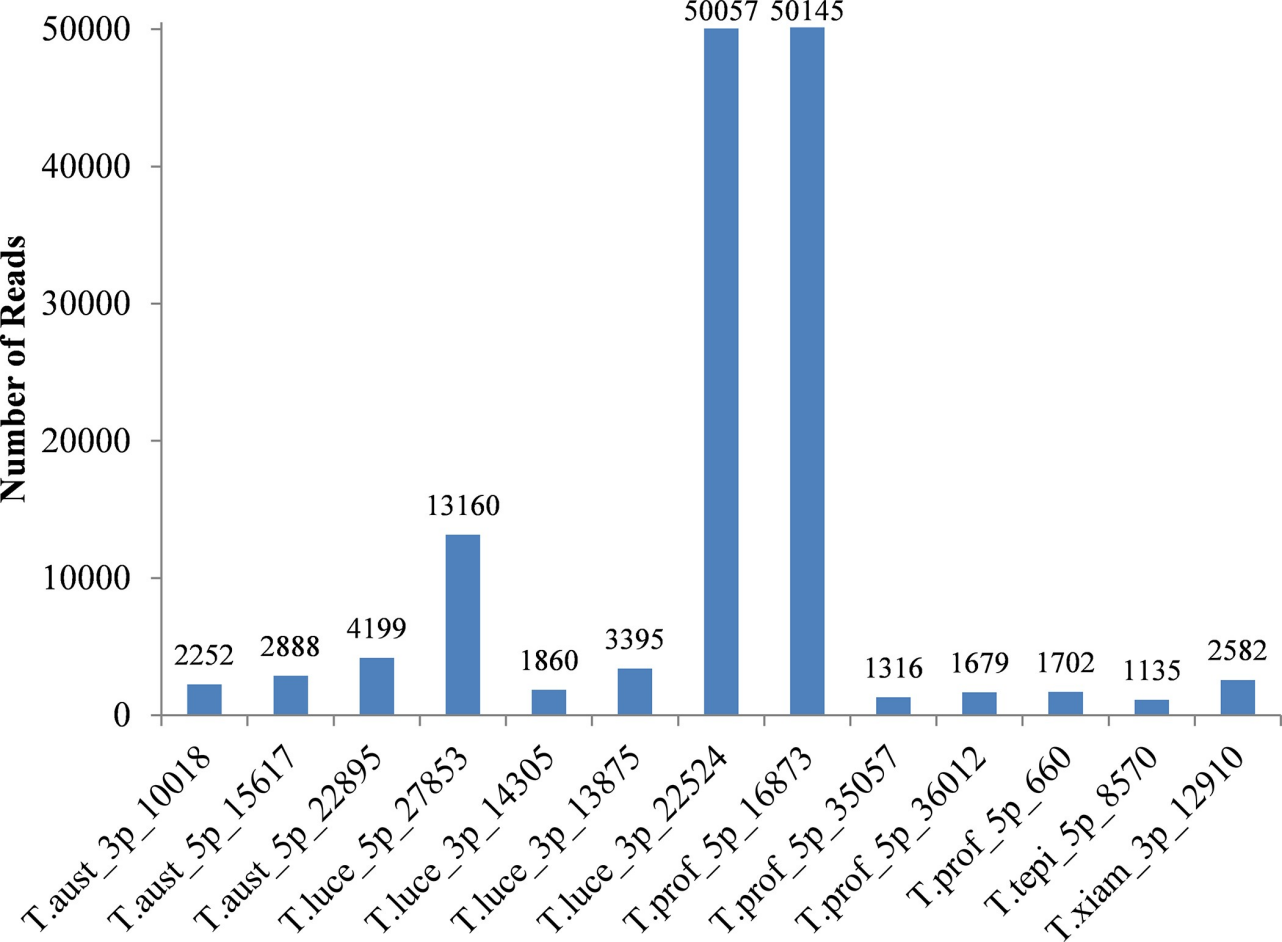
Expression of miRNAs detected in bacteria of the genus *Thalassospira*. Based on number of reads, the high degree of expression of miRNAs was found in *T*. *australica* NP 3b2^T^, *T*. *lucentensis* QMT2^T^, *T*. *profundimaris* WP0211^T^, *T*. *tepidiphila* 1-1B^T^ and *T*. *xiamenensis* M-5^T^.

The identified putative miRNAs are 18–25 nucleotides in length (**Table 2**), which is in the range previously reported for animal and plant miRNAs [30]. These results are also consistent with the length distribution of miRNAs recently detected in *E*. *coli* DH10B and *Streptococcus mutans* ATCC 25175 [8, 9]. Based on the number of read counts, these putative miRNAs appear to have various expression levels among nine libraries, ranging from one to thousands of reads in each library, under the normal growth condition. Among the 86 detected putative miRNAs, the highest expression was found in T.prof_5p_16873, with 50145 reads, while the lowest expression was shared between T.alka_5p_4136 and T.alka_5p_1889 with only one read each being found in the libraries. Of the 86 detected putative miRNAs, thirteen were in high abundance, with over a thousand reads [10] with three miRNAs from *T*. *australica* NP 3b2^T^, four from *T*. *lucentensis* QMT2^T^, four from *T*. *profundimaris* WP0211^T^ and one each from *T*. *tepidiphila* 1-1B^T^ and *T*. *xiamenensis* M-5^T^ being identified. The high degree of expression of these putative miRNAs suggested that they may play specific roles involving in the growth and development processes of these bacteria (**Fig 2**).

### Identification of miRNAs using CID-miRNA method

The 86 putative miRNAs detected in nine *Thalassospira* species using miRDeep method appeared to be rather low in number compared to 400 miRNAs identified in *E*. *coli* DH10B [10] and 900 miRNAs in *Streptococcus mutans* ATCC 25175 [9]. Therefore, an alternative method, CID-miRNA[24] was employed in the expectation of identifying further potential miRNAs from the dataset. CID-miRNA is a web-server developed for the identification of the miRNA precursors based on the secondary structure filter and an algorithm of stochastic context free grammar (SCFG) [24]. The server was first used to predict the potential miRNA precursors in human genome [24] which was later applied in animals such as mouse, zebrafish and sea squirt [31]. Using this method, 449 potential miRNA precursors were identified from over 242 million reads of *T*. *australica* NP 3b2^T^ (77 potential miRNA precursors), *T*. *lucentensis* QMT2^T^ (161), *T*. *profundimaris* WP0211^T^ (78), *T*. *xiamenensis* M-5^T^ (16), *T*. *alkalitolerans* JCM 18968^T^ (21), *T*. *mesophila* JCM 18969^T^ (1), *T*. *povalilytica* Zumi 95^T^ (2), *T*. *tepidiphila* 1-1B^T^ (61) and *T*. *xianhensis* P-4^T^ (32).

In order to identify the mature miRNAs, these precursor sequences were further analysed by employing another web tool, *MatureBayes* (http://mirna.imbb.forth.gr/MatureBayes.html). *MatureBayes* is designed to incorporate to the Naïve Bayes classifier for identifying the putative mature miRNA molecules based on the sequence and structure of the miRNA precursors [25]. This tool provided putative mature miRNA positions in the 5’ and 3’ direction of the miRNA precursors, resulting in 898 putative mature miRNAs in the 9 *Thalassospira* species, comprised of *T*. *australica* NP 3b2^T^ (154 miRNAs), *T*. *lucentensis* QMT2^T^ (322 miRNAs), *T*. *profundimaris* WP0211^T^ (156 miRNAs), *T*. *xiamenensis* M-5^T^ (32 miRNAs), *T*. *alkalitolerans* JCM 18968^T^ (42 miRNAs), *T*. *mesophila* JCM 18969^T^ (2 miRNAs), *T*. *povalilytica* Zumi 95^T^ (4 miRNAs), *T*. *tepidiphila* 1-1B^T^ (122 miRNAs) and *T*. *xianhensis* P-4^T^ (64 miRNAs) **(S2 Table**). These identified putative miRNAs were used for further analysis of miRNA conservation.

### Comparison of bacterial miRNAs identified using miRDeep2 and CID-miRNA

In order to determine whether any miRNAs were detected by both methods, miRNA data generated from miRDeep were BLAST-searched to those produced by CID-miRNA. The results showed that there were no shared putative mature miRNA sequences. miRDeep2 predicts putative miRNAs based on distribution of minimum free energy (MFE) and stability of secondary structure established for nematode, *C*. *elegans*, while CID-miRNA utilised secondary structure filter and stochastic context free grammar trained on human miRNAs [23, 24]. The differences of parameter setting may cause no overlap of putative mature miRNA sequences detected in both methods. Thakur et al [32] also pointed out that application of new parameters improved the accuracy of plant miRNA prediction using miRDeep in compared to the default setting for animals.

**Fig 3:**
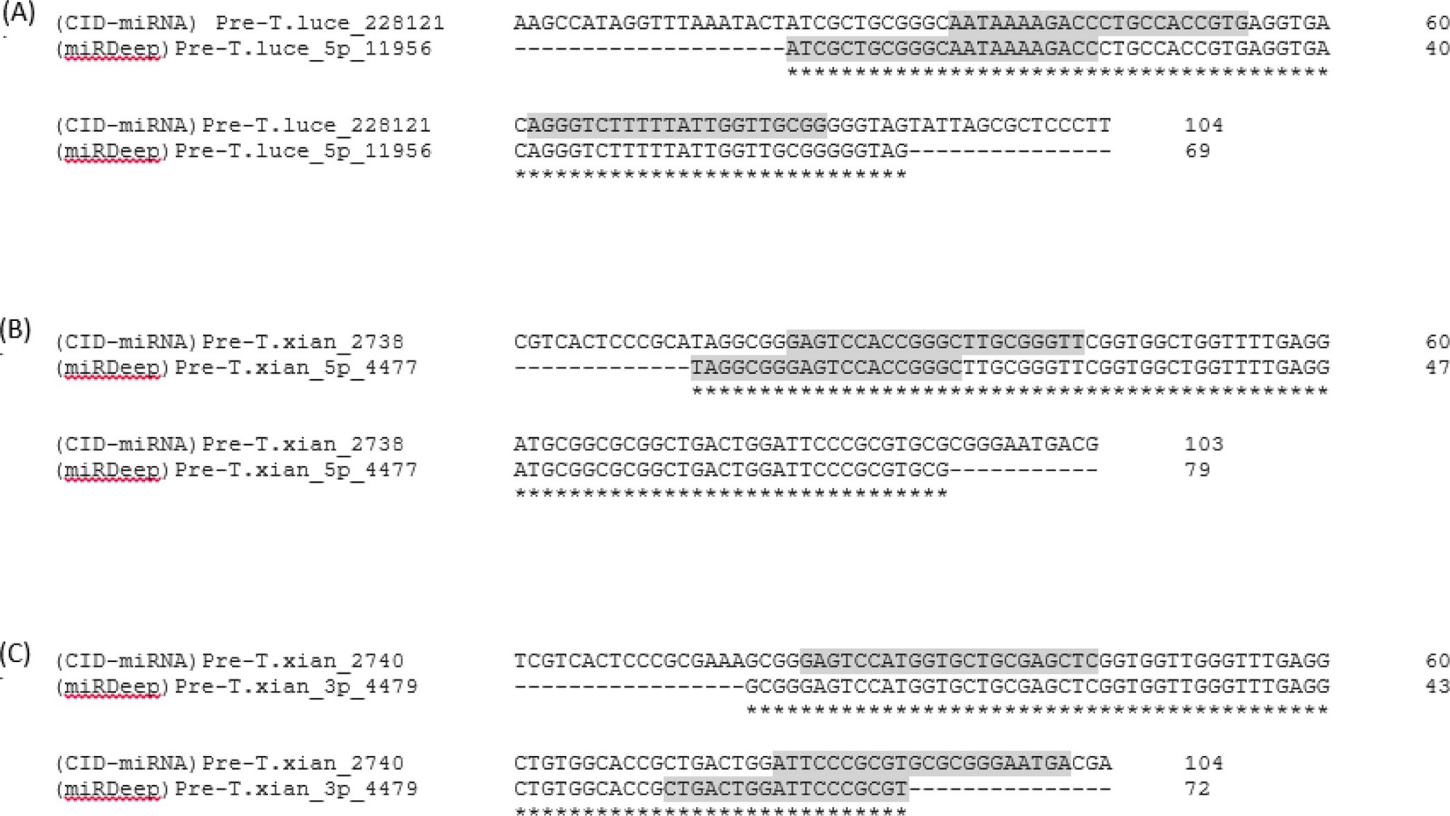
Sharing sequences of potential miRNA precursors identified in both miRDeep2 and CID-miRNA methods. (A) T.luce_228121 precursor from CID-miRNA and T.luce_5p_11956 precursor from miRDeep2. (B) T.xian_2738 precursor from CID-miRNA and T.xian_5p_4477 precursor from miRDeep2. (C) T.xian_2740 precursor from CID-miRNA and T.xian_3p_4479 precursor from miRDeep2. The asterisk (*) indicate that the nucleotides are identical to the top sequence. The putative mature miRNAs are highlighted in grey. (CID-miRNA) indicated the potential precursor detected by CID-miRNA method. (miRDeep2) indicated the potential precursor detected by miRDeep2 method.

Study on miRNA biogenesis showed that one or more mature miRNAs can be produced from one pre-miRNA molecule [33]. These identified putative miRNAs, therefore, were aligned to the potential precursor miRNA sequences generated by both methods. BLAST-search between putative mature miRNA sequences identified from miRDeep2 and the potential precursor miRNAs detected by CID-miRNA was carried out and vice versa. 5 putative mature miRNA sequences identified by CID-miRNA (T.luce_5p_228121, T.luce_3p_228121, T.xian_5p_2738, T.xian_5p_2740 and T.xian_3p_2740) were found to locate in 3 potential precursor miRNAs producing T.luce_5p_11956, T.xian_5p_4477 and T.xian_3p_4479 from miRDeep2. The precursor miRNA sequences producing 5 putative mature miRNAs from CID-miRNA were then aligned to 3 potential precursor miRNAs from miRDeep2 using ClustalW2. The result showed the overlap of 3 precursor miRNAs predicted in both methods in which the putative mature miRNAs identified by each method located at different positions within the precursor sequences (**Fig 3**).

The shared sequences from the same *Thalassospira* species proved the probability of putative miRNAs finding in both methods. Thus, a total of 984 putative miRNA candidates were preliminarily obtained from both methods with default setting together for 9 species (**Table 3**). Further work, such as real time PCR or Northern blot analysis, is needed to verify these putative miRNAs.

**Table 3:** Identification of miRNAs using miRDeep2 and CID-miRNA methods.

Organism	miRDeep2 analysis	CID-miRNA analysis	Total
***T*. *alkalitolerans* JCM 18968**^**T**^	11	42	53
***T*. *australica* NP 3b2**^**T**^	11	154	165
***T*. *lucentensis* QMT2**^**T**^	11	322	333
***T*. *mesophila* JCM 18969**^**T**^	5	2	7
***T*. *povalilytica* Zumi 95**^**T**^	4	4	8
***T*. *profundimaris* WP0211**^**T**^	14	156	170
***T*. *tepidiphila* 1-1B**^**T**^	9	122	131
***T*. *xianhensis* P-4**^**T**^	15	64	79
***T*. *xiamenensis* M-5**^**T**^	6	32	38

### Conservation of miRNAs in the genus *Thalassospira* and miRNAs previously described in *Streptococcus mutans* ATCC 25175 and *E*. *coli* DH10B

Previous studies showed the conservation of some miRNAs across animals or the plant kingdom [34, 35]; however, novel sequences can only be found in a particular species. The conserved and novel miRNAs in bacteria still remain largely unknown, in comparison to intensive studies of miRNAs in eukaryotic organisms and viruses. In this study, 984 putative miRNA candidates of the genus *Thalassospira* were BLAST-searched against those reported for *E*. *coli* DH10B (400 miRNAs) and *S*. *mutans* ATCC 25175 (900 miRNAs) [9, 10] in order to identify any conserved miRNA. However, no conserved sequences could be found without any mismatch or with three and fewer nucleotide substitution. *E*. *coli* is an enteric bacterium commonly found in low intestine of humans and animals [36], *S*. *mutans* is an oral pathogen that causes human dental caries [37], while bacteria of the *Thalassospira* genus are environmental bacteria [14]. The differences in their characteristics may influence the low degree of conservation of miRNAs among these bacteria. The results obtained in this study is in agreement with previous studies that reported the lack of significant sequence similarity in non-coding RNA homologues of different bacterial species [38, 39].

In order to identify any conserved miRNAs among species of the genus *Thalassospira*, the putative miRNA sequences obtained were compared. It appears that the nine species shared 57 common putative miRNAs. Among these, *T*. *profundimaris* WP0211^T^ and *T*. *tepidiphila* 1-1B^T^ had the highest number of conserved miRNAs, with 45 sequences presenting in both species. Five putative miRNAs in *T*. *alkalitolerans* JCM 18968^T^ were also found in the *T*. *lucentensis* QMT2^T^, while *T*. *xianhensis* P-4^T^ and *T*. *xiamenensis* M-5^T^ shared 4 conserved sequences. One sequence was shared between *T*. *australica* NP 3b2^T^ and *T*. *tepidiphila* 1-1B^T^, *T*. *lucentensis* QMT2^T^ and *T*. *tepidiphila* 1-1B^T^ and *T*. *lucentensis* QMT2^T^ and *T*. *mesophila* JCM 18969^T^ (**Table 4**). As seen from the data, *T*. *lucentensis* QMT2^T^ has identical miRNA sequences as *T*. *alkalitolerans* JCM 18968^T^ (5 sequences), *T*. *tepidiphila* 1-1B^T^ (1) and *T*. *mesophila* JCM 18969^T^ (1), while *T*. *tepidiphila* 1-1B^T^ also shared sequences with *T*. *profundimaris* WP0211^T^ (45) and *T*. *australica* NP 3b2^T^ (1).

**Table 4:** Conserved miRNAs in different species of bacteria of the genus *Thalassospira*.

miRNA sequence	*T*. *alkalitolerans* JCM 18968^T^	*T*. *australica* NP 3b2^T^	*T*. *lucentensis* QMT2^T^	*T*. *mesophila* JCM 18969^T^	*T*. *profundimaris* WP0211^T^	*T*. *tepidiphila* 1-1B^T^	*T*. *xianhensis* P-4^T^	*T*. *xiamenensis* M-5^T^
TTAATCCGGACCCATTAATTAT	T. alka_5p_4942		T. luce_5p_520914					
CATAATTAATGTGTTCGGAACT	T. alka_3p_4942		T. luce_3p_520914					
ATCAGGTCGAAGCCATGACCAT	T. alka_5p_1819		T. luce_5p_218949					
TCTGGCATCGGCGTTTCTATCG	T. alka_5p_4684		T. luce_5p_498538					
CGTCGTCTGATCCGCTTTGCCA	T. alka_3p_4684		T. luce_3p_498538					
AGCGACAACGCCGGTGGGATCA					T. profu_5p_37885	T. tepi_5p_24769		
TGCCACCGGCGTTGTTGTCTTC					T. profu_3p_37885	T. tepi_3p_24769		
AAGAAGCAGCGTCGGCCAGCCA					T. profu_5p_14495	T. tepi_5p_9431		
GCCGGCGCTGCCTCAACTCGTT					T. profu_3p_14495	T. tepi_3p_9431		
ATCAAAAAGGCGGAGCTGATTT					T. profu_5p_9602	T. tepi_5p_6592		
CTCCGCCTTTTTTTTGTTCGAG					T. profu_3p_9602	T. tepi_3p_6592		
AATCATCGATCCGTTGATCTTC					T. profu_5p_49438	T. tepi_5p_33046		
AACTTTGTGCAGCTGTTTGGTC					T. profu_3p_49438	T. tepi_3p_33046		
CGCGGCGGTGGCGTTGCCGAAC					T. profu_5p_45214	T. tepi_5p_29936		
AACGTGATGGCGTCATGCACCG					T. profu_5p_45214	T. tepi_3p_29936		
CGGTTGCAATTGCGACCACCAC					T. profu_5p_29989	T. tepi_5p_19433		
CCTTATAGCGGAATGCGCCCTG					T. profu_3p_29989	T. tepi_3p_19433		
AACAAAACCCGCAAGGCCAATG					T. profu_5p_41148	T. tepi_5p_26936		
TTGCGGGTTTTGCTGTGATGTT					T. profu_3p_41148	T. tepi_3p_26936		
GGGGGGAAAAGTTCCCTTGCCG					T. profu_5p_54785	T. tepi_5p_36487		
TTGCCGAACGGCTGAAAGAGCT					T. profu_3p_54785	T. tepi_3p_36487		
CAATCTGTTGCAGTGCCTGATC					T. profu_5p_30109	T. tepi_5p_19555		
CTGATCTGCTTCGTTACGGATA					T. profu_3p_30109	T. tepi_3p_19555		
TGCCTATCGCGTCGACGAGGTG					T. profu_5p_7879	T. tepi_5p_5471		
TGTCGAGGCGGCTGGTCTGCGT					T. profu_3p_7879	T. tepi_3p_5471		
CACCGATGTCGAAAGATCTTCG					T. profu_5p_778	T. tepi_5p_572		
TTCCATATCGTGTCGTTATTCA					T. profu_3p_778	T. tepi_3p_572		
AATTGTATGTGCAATAATGCGA					T. profu_5p_32260	T. tepi_5p_20938		
GCGTTCGGAGGATTGCACATGC					T. profu_3p_32260	T. tepi_3p_20938		
CTCATGAGTAATGTGTTCGGAA					T. profu_3p_3800	T. tepi_3p_2570		
TGTGATGGTTTCTTCTATCGCA					T. profu_5p_19048	T. tepi_5p_11782		
GTCGGTGGCGGTAACACCGCGG					T. profu_3p_19048	T. tepi_3p_11782		
TTTCAACAACGCCCGTTGATTG					T. profu_5p_38661	T. tepi_5p_25251		
ATTGAAATCCCCCGCCTAAACC					T. profu_3p_38661	T. tepi_3p_25251		
ATTTTGTACCTGATGAAACGGC					T. profu_5p_42425	T. tepi_5p_27927		
CGTTTTGTTAGGTGTTAACCTG					T. profu_3p_42425	T. tepi_3p_27927		
TATGCCAACAATCCGACCGGGT					T. profu_5p_24235	T. tepi_5p_15757		
GCGGTCTGGATGTTGGCCTGCC					T. profu_3p_24235	T. tepi_3p_15757		
AGCAAAAGCTGCCTAATTAAGG					T. profu_5p_21694	T. tepi_5p_13698		
CTTCTGCTTTACAGACAGAATT					T. profu_3p_21694	T. tepi_3p_13698		
TGTCTTTTTCTGACGTTTTTTC					T. profu_5p_49945	T. tepi_5p_33271		
CGTTTTTTCTCAAAAAAGGGTT					T. profu_3p_49945	T. tepi_3p_33271		
TTGTCTGTCAAACAGGCAAGGA					T. profu_5p_17170	T. tepi_5p_11264		
AAGGATTGCGGTCGGCCTTACT					T. profu_3p_17170	T. tepi_3p_11264		
TGACGCAGAGGCTTTCTCTCAT					T. profu_5p_51572	T. tepi_5p_34376		
AGGTGGCCTTTGGATCACCCGG					T. profu_3p_51572	T. tepi_3p_34376		
GTCGGCGTTGTCGCGCTGTTCA					T. profu_5p_12216	T. tepi_5p_8405		
TTCAAGGAGCCGCTGCATGTTG					T. profu_3p_12216	T. tepi_3p_8405		
AGACGTGACCTTCGGGTCGCGT							T. xian_5p_16668	T. xiam_5p_26826
CGTCTTTTTTATTGTCTGGTGG							T. xian_3p_16668	T. xiam_3p_26826
CAATTAAAAACCCCCTCAGGCG							T. xian_5p_6822	T. xiam_5p_11702
AGGGGTTTTTTAATTGGTAGCC							T. xian_3p_6822	T. xiam_3p_11702
CAATTTAAACATTGTCACCATG		T. aust_3p_49513				T. tepi_3p_22906		
ATAATGACGTCCGTTGCGAC			T.luce_5p_10500			T.tepi_5p_8152		
TCACAGTCGAGACGCTCTCTCACC			T.luce_3p_22524	T.meso_3p_3562				
GTCCGGTGGTCTGGGCACCATG					T.prof_5p_36012	T.tepi_5p_22622		
ATCCTGCCCCCGCAACCA					T.prof_5p_14768	T.tepi_5p_8570		

A comparative analysis of 16S rRNA sequence similarities revealed that the highest number of common miRNAs shared by the phylogenetically close species; *e*.*g*., *T*. *tepidiphila* 1-1B^T^ were found to be phylogenetically closely related to *T*. *profundimaris* WP0211^T^ (99.3% sequence similarity); *T*. *xianhensis* P-4^T^ and *T*. *xiamenensis* M-5^T^ also shared 99.3% 16S rRNA similarity, while *T*. *mesophila* JCM 18969^T^ and *T*. *alkalitolerans* JCM 18968^T^ shared 95.3% and 94.9% of 16S rRNA sequence similarity, respectively, with *T*. *lucentensis* QMT2^T^ [14]. Conserved miRNA sequences found in these species may indicate a close genetic relationship and that these miRNAs have a similar role in the regulation of the growth and development of bacteria. It is interesting to note that two species, *T*. *australica* NP 3b2^T^ and *T*. *tepidiphila* 1-1B^T^, which are capable of hydrocarbon degradation [12, 14], shared one conserved miRNAs and the potential targets of this putative miRNA needs to be investigated for any role related to this process. In addition, *T*. *alkalitolerans* JCM 18968^T^, *T*. *lucentensis* QMT2^T^ and *T*. *xianhensis* P-4^T^ were also found to have the same sequence presented in different genomic locations (**Table 5**). These miRNAs can have an influence on their expression and function at different genomic locations [40]. It will be of great interest to identify the target mRNA of these miRNAs and investigate their roles and the mechanisms of gene regulation in the physiology of these unique environmental bacteria.

**Table 5:** miRNAs present in different locations of the same *Thalassospira* species.

miRNA sequence	*T*. *alkalitolerans* JCM 18968^T^		*T*. *lucentensis* QMT2^T^		*T*. *xianhensis* P-4^T^	
AATTAATGGGTCCTGACC	T.alka_3p_6323	T.alka_3p_6324				
ATTCCCGCGTGCGCGGGAATGA					T. xian_3p_2740	T. xian_3p_2738
TTCGGTGCTCACGTACTTTTAG			T. luce_5p_528656	T. luce_5p_528636		
TGCGCTCCGATGCGCGTGAACC			T. luce_3p_528656	T. luce_3p_528636		
TCGTGCGTCAGCTTGGCGTGAC			T. luce_5p_389406	T. luce_5p_389408		
TCACCCGACCTGACCATGGTCG			T. luce_3p_389406	T. luce_3p_389408		

## Conclusions

Over 242 million reads of 18 to 33 nucleotides length were generated from nine bacterial species using high-throughput sequencing technology. Using miRDeep2 and CID-miRNA analyses, a total of 984 putative miRNAs were eventually identified, with typical miRNA length of 19–25 nucleotides. Compared to other species, these detected putative miRNAs were not conserved to those reported in *E*. *coli* DH10B and *S*. *mutans* ATCC 25175. This study presents the first comprehensive list of computationally identified miRNAs in 9 bacterial species of the genus *Thalassospira* without experimental verification. The further work, however, is needed to validate these candidates experimentally. In addition, further identification of miRNA targets will provide insights into the fundamental functions of miRNAs in the physiology of these bacteria.

## Supporting information

S1 Fig Phylogenetic tree of valid species of the genus *Thalassospira*. (DOCX)

S1 Table Whole genome sequences of bacteria of the genus *Thalassospira* available in GenBank. (DOCX)

S2 Table miRNAs retrieved from bacteria of the genus *Thalassospira* as identified by CID-miRNA analysis. (DOCX)
