# Supplementary material for: Computational prediction of microRNAs in marine bacteria of the genus Thalassospira
Source: PLoS One. 2019 Mar 12;14(3):e0212996. doi: 10.1371/journal.pone.0212996 (PMC6413936; doi:10.1371/journal.pone.0212996)
Supplement: S1 Fig — (DOCX) [file pone.0212996.s001.docx]

*Thalassospira profundimaris* WP0211^T^ (AY186195)

*Thalassospira tepidiphila* 1-1B^T^ (AB265822)

*Thalassospira povalilytica* Zumi 95^T^ (AB548215)

*Thalassospira australica* NP3b2^T^ (KU553304)

*Thalassospira xiamenensis* M-5^T^ (AY189753)

*Thalassospira xianhensis* P-4^T^ (EU017546)

*Thalassospira lucentensis* QMT2^T^ (AF358664)

*Thalassospira alkalitolerans* JCM 18968^T^ (AB786710)

*Thalassospira mesophila* JCM 18969^T^ (AB786711)

*Azorhizobium caulinodans* LMG 6465^T^ (X67221)

84

84

99

94

99

97

84

0.020

**S1 Fig.** **Phylogenetic tree of valid species of the genus *Thalassospira* based on the 16S rRNA gene sequence similarities from the neighbour-joining (NJ) phylogenetic tree**. The *Azorhizobium caulinodans* LMG 6465^T^ (X67221) was used as the outgroup in the analysis. The numbers given at the branching points are percentage bootstrap values based on 1000 replications, with only values above 50% being shown. The scale bar represents 0.02 substitutions per nucleotide position.
