# Supplementary material for: Computational prediction of microRNAs in marine bacteria of the genus Thalassospira
Source: PLoS One. 2019 Mar 12;14(3):e0212996. doi: 10.1371/journal.pone.0212996 (PMC6413936; doi:10.1371/journal.pone.0212996)
Supplement: S1 Table — (DOCX) [file pone.0212996.s002.docx]

**S1** **Table. Whole genome sequences of bacteria of the genus *Thalassospira* available in GenBank.**

| **Samples** | **References** | **GenBank accession numbers** |
| --- | --- | --- |
| *T. australica* NP 3b2^T^ | *T. australica* NP 3b2^T^ | JRJE00000000.1 |
| *T. lucentensis* QMT2^T^ | *T. lucentensis* QMT2^T^ | ATWN00000000.1 |
| *T. alkalitolerans* JCM 18968^T^ |  |  |
| *T. mesophila* JCM 18969^T^ |  |  |
| *T. profundimaris* WP0211^T^ | *T. profundimaris* WP0211^T^ | AMRN00000000.1 |
| *T. tepidiphila* 1-1B^T^ |  |  |
| *T. povalilytica* Zumi 95^T^ |  |  |
| *T. xiamenensis* M-5^T^ | *T. xiamenensis* M-5^T^ | CP004388 |
| *T. xianhensis* P-4^T^ |  |  |
