# Supplementary material for: Computational prediction of microRNAs in marine bacteria of the genus Thalassospira
Source: PLoS One. 2019 Mar 12;14(3):e0212996. doi: 10.1371/journal.pone.0212996 (PMC6413936; doi:10.1371/journal.pone.0212996)
Supplement: S2 Table — (DOCX) [file pone.0212996.s003.docx]

**S2 Table.** **miRNAs retrieved from bacteria of the genus *Thalassospira* as identified by CID-miRNA analysis**.

| **miRNA*** | **Location** | **Sequence** |
| --- | --- | --- |
| ***T. alkalitolerans* JCM 18968^T^** | | |
| T. alka_5p_4279 | ATWN01000008.1_4279 | CAAGGTGGTAAGCGGTGGTTAT |
| T. alka_3p_4279 |  | ATAACCGCCGCTTTCTCCTTTC |
| T. alka_5p_3838 | ATWN01000007.1_3838 | CGAAACGGATCACCGTTTCAGG |
| T. alka_3p_3838 |  | AGGGCCTTTGAATAATGGTCGG |
| T. alka_5p_329 | ATWN01000001.1_329 | GGATTAGGAGGCTGGCAACAAC |
| T. alka_3p_329 |  | TGCCAGCCTCCTTTTTAGGTTC |
| T. alka_5p _582 | ATWN01000001.1_582 | CAAAATTCATCATGTTCGACCT |
| T. alka_3p _582 |  | CGGGGTCGATGATTTGCTTTTT |
| T. alka_5p_1519 | ATWN01000002.1_1519 | AAGGGTTGGTTCCGACCTATCT |
| T. alka_3p _1519 |  | TATCTTGAACAGTCGATGCAGG |
| T. alka_5p_333 | ATWN01000001.1_333 | TTGTTACTTCAATGATCCGGTT |
| T. alka_3p _333 |  | TTCGGGGCTGGATCGTGTGCTG |
| T. alka_5p_3811 | ATWN01000007.1_3811 | GCTGGTATCGGCCTTTGCCGGG |
| T. alka_3p_3811 |  | CCGGGTTTGACCGGATGAAGGC |
| T. alka_5p_3149 | ATWN01000005.1_3149 | TCATCCTGCTTGAAGCGCACCG |
| T. alka_3p_3149 |  | GGCGCTTCGGGCCGGAATGGCG |
| T. alka_5p_4942 | ATWN01000010.1_4942 | TTAATCCGGACCCATTAATTAT |
| T. alka_3p_4942 |  | CATAATTAATGTGTTCGGAACT |
| T. alka_5p_2354 | ATWN01000004.1_2354 | CAGTGTGTTCGAATGATCCGGT |
| T. alka_3p_2354 |  | CGATTGATCTGTTTGAAGACAT |
| T. alka_5p_4120 | ATWN01000007.1_4120 | CCTAATCAGCAGTTATTTCCTG |
| T. alka_3p_4120 |  | AAAACCCCGCCAGAATGTTCTG |
| T. alka_5p_2265 | ATWN01000004.1_2265 | AGTTGCGCATCGATATGGAAGA |
| T. alka_3p _2265 |  | TTGAGCTTCTGATACCGTATGC |
| T. alka_5p_5955 | ATWN01000015.1_5955 | CAAAACGCCTGCTGATTGACTG |
| T. alka_3p _5955 |  | TCATTCGCTGCGTAAACTTGGC |
| T. alka_5p_1023 | ATWN01000002.1_1023 | TTTATCTGTGTCGGGTGTGGAA |
| T. alka_3p_1023 |  | GACATCACACCGATGGAAAATC |
| T. alka_5p_5176 | ATWN01000011.1_5176 | TTTGTTTTTGATACAAGCTAAA |
| T. alka_3p_5176 |  | TTGCATTAAGAACGGGGTCACG |
| T. alka_5p_903 | ATWN01000001.1_903 | CGTTCGCTCTGCATGCAGCCGA |
| T. alka_3p_903 |  | TGTTTGATGAGCCGACGTCCGC |
| T. alka_5p_3591 | ATWN01000006.1_3591 | AGATGTCCGCATCAGCGCCCTT |
| T. alka_3p _3591 |  | CGCCCTTGGCAAGAAAGGCCGT |
| T. alka_5p_1819 | ATWN01000003.1_1819 | ATCAGGTCGAAGCCATGACCAT |
| T. alka_3p _1819 |  | ATGACCATGGCCGACAAAATCG |
| T. alka_5p_5116 | ATWN01000010.1_5116 | TTGAAAGATATATATCTTCCTG |
| T. alka_3p _5116 |  | CCGCACAAATCCGGTCAACCAT |
| T. alka_5p_2932 | ATWN01000005.1_2932 | AGGCGGCAAACTTGCCGCCTCA |
| T. alka_3p_2932 |  | CTCATTTTTTGATTGTTGTATT |
| T. alka_5p_4684 | ATWN01000009.1_4684 | TCTGGCATCGGCGTTTCTATCG |
| T. alka_3p_4684 |  | CGTCGTCTGATCCGCTTTGCCA |
| ***T. mesophila* JCM 18969^T^** | | |
| T. meso_5p_159 | ATWN01000001.1_159 | CCGGGCTGTTATCCAACTCCGG |
| T. meso_3p_159 |  | TACAATTGGCGTTAACCCCGCT |
| ***T. povalilytica* Zumi 95^T^** | | |
| T. pova_5p_2290 | AMRN01000009.1_2290 | TCCGGGTCGGGTAAATCGGTTT |
| T. pova_3p_2290 |  | GGTCTGATTGATTTTCCCGGTC |
| T. pova_5p_489 | AMRN01000001.1_489 | TTCATCCAGCCGATGCGCGCGA |
| T. pova_3p_489 |  | CGCGCGACACAGTTAAAGATCC |
| ***T. profundimaris* WP0211^T^** | | |
| T. profu_5p_28449 | AMRN01000004.1_28449 | TTATGCGAAATTGAGAAGCGTT |
| T. profu_3p_28449 |  | ATTTGTTTCAGGCATAGGACAT |
| T. profu_5p_49903 | AMRN01000010.1_49903 | CGAGGCCCCGATGGGCCTCCCC |
| T. profu_3p_49903 |  | CTCCCCAGATGGGGAAAGCCCC |
| T. profu_5p_52536 | AMRN01000011.1_52536 | AAAAAAACGCCTGACCGGTTTT |
| T. profu_3p_52536 |  | TCAGGCGTTTTTTTGTTCGTGT |
| T. profu_5p_56451 | AMRN01000014.1_56451 | AAAGCTTCCTCTTTGAAAATCT |
| T. profu_3p_56451 |  | TATCTTTTTCAAAGGAAGTCAT |
| T. profu_5p_37885 | AMRN01000006.1_37885 | AGCGACAACGCCGGTGGGATCA |
| T. profu_3p_37885 |  | TGCCACCGGCGTTGTTGTCTTC |
| T. profu_5p_32508 | AMRN01000005.1_32508 | ACCCCCGGTCGTTAGGCCGGGG |
| T. profu_3p_32508 |  | GGGTGTTTTTTTGCTATAGTTC |
| T. profu_5p_14495 | AMRN01000002.1_14495 | AAGAAGCAGCGTCGGCCAGCCA |
| T. profu_3p_14495 |  | GCCGGCGCTGCCTCAACTCGTT |
| T. profu_5p_40748 | AMRN01000007.1_40748 | TCGGTTGGTAAAATCGCCGATA |
| T. profu_3p_40748 |  | CGATATCTATGCCCACCGTGGG |
| T. profu_5p_57479 | AMRN01000017.1_57479 | TACCTCGATCCATATCGAGGAA |
| T. profu_3p_57479 |  | AGGAATTCGAAGTCATGGCCCG |
| T. profu_5p_5865 | AMRN01000001.1_5865 | TGGATTAAAAAAAAGCGCCGCC |
| T. profu_3p_5865 |  | CGGCGCTTTTTTTTGTTGGCTG |
| T. profu_5p_24901 | AMRN01000003.1_24901 | CCGAAGAGGCCCAGGTGCGGTT |
| T. profu_3p_24901 |  | ATTCCCGCCTGACGATCTTTGT |
| T. profu_5p_17082 | AMRN01000002.1_17082 | CGGGGGTGTCAGCTTTGCCGGG |
| T. profu_3p_17082 |  | TTCGGACCTGATGGTCCCGCCA |
| T. profu_5p_9602 | AMRN01000001.1_9602 | ATCAAAAAGGCGGAGCTGATTT |
| T. profu_3p_9602 |  | CTCCGCCTTTTTTTTGTTCGAG |
| T. profu_5p_49438 | AMRN01000010.1_49438 | AATCATCGATCCGTTGATCTTC |
| T. profu_3p_49438 |  | AACTTTGTGCAGCTGTTTGGTC |
| T. profu_5p_33610 | AMRN01000005.1_33610 | CTGAAAACCGAAAAAGGTCCAT |
| T. profu_3p_33610 |  | CATTTCCGGATCTTTTTTCTGT |
| T. profu_5p_56952 | AMRN01000015.1_56952 | AATTTACCCCTGTCCCGTCCAA |
| T. profu_3p_56952 |  | ATGAGATAGGGGCTGATCTCGA |
| T. profu_5p_45214 | AMRN01000008.1_45214 | CGCGGCGGTGGCGTTGCCGAAC |
| T. profu_3p_45214 |  | AACGTGATGGCGTCATGCACCG |
| T. profu_5p_20856 | AMRN01000003.1_20856 | TCGATCAATATCTACAGCCAGA |
| T. profu_3p_20856 |  | TGATGTTGGTCGGGCTGATGGC |
| T. profu_5p_3073 | AMRN01000001.1_3073 | TTCTGTTTTTGGATCGGCCTGA |
| T. profu_3p_3073 |  | ATCGGCCTGATTGCTGTTATGG |
| T. profu_5p_29989 | AMRN01000004.1_29989 | CGGTTGCAATTGCGACCACCAC |
| T. profu_3p_29989 |  | CCTTATAGCGGAATGCGCCCTG |
| T. profu_5p_17078 | AMRN01000002.1_17078 | CTCCAGCTGTCGGAGACAGGGC |
| T. profu_3p_17078 |  | TGCCTCCGAACAGTCTGGGAGA |
| T. profu_5p_43277 | AMRN01000008.1_43277 | CCGGGAAGGATATTCTTCCCGG |
| T. profu_3p_43277 |  | CGGCCTTTTTGATGTGTTGATG |
| T. profu_5p_41148 | AMRN01000007.1_41148 | AACAAAACCCGCAAGGCCAATG |
| T. profu_3p_41148 |  | TTGCGGGTTTTGCTGTGATGTT |
| T. profu_5p_54785 | AMRN01000013.1_54785 | GGGGGGAAAAGTTCCCTTGCCG |
| T. profu_3p_54785 |  | TTGCCGAACGGCTGAAAGAGCT |
| T. profu_5p_7550 | AMRN01000001.1_7550 | GTGATCTTGAGCTTGATCACGG |
| T. profu_3p_7550 |  | AAGGTCAATGTCCATGGCGGGG |
| T. profu_5p_17011 | RN01000002.1_17011 | TATGATGATGCGCGACAATTTT |
| T. profu_3p_17011 |  | TAGTTCATAATCATAACGGCGA |
| T. profu_5p_33954 | AMRN01000005.1_33954 | GAAAACGATGGGGAGTGGGCGC |
| T. profu_3p_33954 |  | GTCCACTTCCGCTGCGCCGTTT |
| T. profu_5p_44623 | AMRN01000008.1_44623 | TGAATCCAATGCAAGTGATGGT |
| T. profu_3p_44623 |  | ACCATAATTTTGCTGGGCTTGG |
| T. profu_5p_29018 | AMRN01000004.1_29018 | CACCGGAACGGCATCCCGGTGC |
| T. profu_3p_29018 |  | CGGTGCGTTTGTTGGTTTGGCG |
| T. profu_5p_43344 | AMRN01000008.1_43344 | ATCTGTTTTGGGGCGATCCGAA |
| T. profu_3p_43344 |  | CGAACTTTGTTCCTGCCATGAC |
| T. profu_5p_30109 | AMRN01000004.1_30109 | CAATCTGTTGCAGTGCCTGATC |
| T. profu_3p_30109 |  | CTGATCTGCTTCGTTACGGATA |
| T. profu_5p_7879 | AMRN01000001.1_7879 | TGCCTATCGCGTCGACGAGGTG |
| T. profu_3p_7879 |  | TGTCGAGGCGGCTGGTCTGCGT |
| T. profu_5p_43079 | AMRN01000008.1_43079 | AAGAACACCGTTTGATCATCGT |
| T. profu_3p_43079 |  | CGAAGACATGAAAACGCTGTTG |
| T. profu_5p_778 | AMRN01000001.1_778 | CACCGATGTCGAAAGATCTTCG |
| T. profu_3p_778 |  | TTCCATATCGTGTCGTTATTCA |
| T. profu_5p_28659 | AMRN01000004.1_28659 | CGATTTCAATCTCGATGAAGTT |
| T. profu_3p_28659 |  | AAGTTTTATTTTGATAGCGCAG |
| T. profu_5p_57432 | AMRN01000017.1_57432 | ACTACTTCACCGGTCCGACCGC |
| T. profu_3p_57432 |  | CTGATCCGGCTTCGGTCGCAAA |
| T. profu_5p_11482 | AMRN01000001.1_11482 | TTTGGCGGTTTCTCGCCCGAGG |
| T. profu_3p_11482 |  | CGAGGCATTGGCTGGCCGTGTC |
| T. profu_5p_14407 | AMRN01000002.1_14407 | TTGCGTCCGTTTATTGGCGAGG |
| T. profu_3p_14407 |  | AGCGTGTCATGGCCTTCATGCC |
| T. profu_5p_32260 | AMRN01000005.1_32260 | AATTGTATGTGCAATAATGCGA |
| T. profu_3p_32260 |  | GCGTTCGGAGGATTGCACATGC |
| T. profu_5p_3800 | AMRN01000001.1_3800 | ACCCGATGCTTACGCCGGACCC |
| T. profu_3p_3800 |  | CTCATGAGTAATGTGTTCGGAA |
| T. profu_5p_38494 | AMRN01000007.1_38494 | TTCAATCTTGCCCGCTATCTTG |
| T. profu_3p_38494 |  | ACATGTGGTCAAGGTTGTTCGA |
| T. profu_5p_51013 | AMRN01000010.1_51013 | CAGCATGCTGAATGCGGCGCGC |
| T. profu_3p_51013 |  | ATCGCTTTCGGCTTGGTGTAGT |
| T. profu_5p_12371 | AMRN01000002.1_12371 | AATGGGTTGATGTCGGGGCACG |
| T. profu_3p_12371 |  | AATAGCCCACGGCATCACTCAT |
| T. profu_5p_33977 | AMRN01000005.1_33977 | CATCAGAGATGCAGGGCCTATT |
| T. profu_3p_33977 |  | CAGGGCCTATTTAATTGGTAGC |
| T. profu_5p_45949 | AMRN01000008.1_45949 | CTGGGCCAACGGGCTTGTCATT |
| T. profu_3p_45949 |  | GTCATTCTGATTGCCTGCATGA |
| T. profu_5p_19048 | AMRN01000008.1_45949 | TGTGATGGTTTCTTCTATCGCA |
| T. profu_3p_19048 |  | GTCGGTGGCGGTAACACCGCGG |
| T. profu_5p_38661 | AMRN01000007.1_38661 | TTTCAACAACGCCCGTTGATTG |
| T. profu_3p_38661 |  | ATTGAAATCCCCCGCCTAAACC |
| T. profu_5p_42425 | AMRN01000007.1_42425 | ATTTTGTACCTGATGAAACGGC |
| T. profu_3p_42425 |  | CGTTTTGTTAGGTGTTAACCTG |
| T. profu_5p_55792 | AMRN01000014.1_55792 | TCGTGCCGATGGCTCGGCGATC |
| T. profu_3p_55792 |  | ACGGCGAGCCGATCGGCACGCG |
| T. profu_5p_15172 | AMRN01000002.1_15172 | ATTTTGCAATCGCTTATCGCTT |
| T. profu_3p_15172 |  | AGCGATCCGCGATTTGTTGAGC |
| T. profu_5p_54600 | AMRN01000012.1_54600 | TTTCCACTGCTGGAAACGCGGC |
| T. profu_3p_54600 |  | ACGCGGCGGTCATGGCTGGATG |
| T. profu_5p_18009 | AMRN01000002.1_18009 | GTTTGGCGACCCTGATCGACCG |
| T. profu_3p_18009 |  | ACCGGTCCATCTCGGCCGCCGA |
| T. profu_5p_44551 | AMRN01000008.1_44551 | CAGGGCATTTCTGCCCTAGCCT |
| T. profu_3p_44551 |  | CTAGCCTTTCGGATGGTTTGCG |
| T. profu_5p_24235 | AMRN01000003.1_24235 | TATGCCAACAATCCGACCGGGT |
| T. profu_3p_24235 |  | GCGGTCTGGATGTTGGCCTGCC |
| T. profu_5p_44441 | AMRN01000008.1_44441 | AGTGTACTCAGTGAGTATGCTC |
| T. profu_3p_44441 |  | ATGCTCATTTAAATCGGAGGCG |
| T. profu_5p_18810 | AMRN01000002.1_18810 | CGGCGATCATGATTGCCGCGGC |
| T. profu_3p_18810 |  | CGCGGCCGCCGGCGCCTATGTG |
| T. profu_5p_41532 | AMRN01000007.1_41532 | TCTCGGGGACGGGTGCGGAACT |
| T. profu_3p_41532 |  | AAGTCCGCGCAGCCTACCTCGA |
| T. profu_5p_44043 | AMRN01000008.1_44043 | GGGGGAATGCGTTCCCTATGCC |
| T. profu_3p_44043 |  | ATGCCCGGTATAATGAAACGGT |
| T. profu_5p_21694 | AMRN01000003.1_21694 | AGCAAAAGCTGCCTAATTAAGG |
| T. profu_3p_21694 |  | CTTCTGCTTTACAGACAGAATT |
| T. profu_5p_49945 | AMRN01000010.1_49945 | TGTCTTTTTCTGACGTTTTTTC |
| T. profu_3p_49945 |  | CGTTTTTTCTCAAAAAAGGGTT |
| T. profu_5p_17170 | AMRN01000002.1_17170 | TTGTCTGTCAAACAGGCAAGGA |
| T. profu_3p_17170 |  | AAGGATTGCGGTCGGCCTTACT |
| T. profu_5p_51572 | AMRN01000011.1_51572 | TGACGCAGAGGCTTTCTCTCAT |
| T. profu_3p_51572 |  | AGGTGGCCTTTGGATCACCCGG |
| T. profu_5p_56542 | AMRN01000015.1_56542 | CGATCGCCGTCACTTCGGCCTT |
| T. profu_3p_56542 |  | TGCCGAGGGCCCGCGTGCGGTC |
| T. profu_5p_30378 | AMRN01000005.1_30378 | CGGATGAATTTTCCGTCCTGGA |
| T. profu_3p_30378 |  | TCCTGGATGGCCACGAAATAGA |
| T. profu_5p_40629 | AMRN01000007.1_40629 | TTGTTGTAATACAGAAGCGGGG |
| T. profu_3p_40629 |  | CAGAAGCGGGGTCGAGCTGTTG |
| T. profu_5p_12216 | AMRN01000002.1_12216 | GTCGGCGTTGTCGCGCTGTTCA |
| T. profu_3p_12216 |  | TTCAAGGAGCCGCTGCATGTTG |
| T. profu_5p_15457 | AMRN01000002.1_15457 | CGCAAGGAAAAGCCCCGCAGAC |
| T. profu_3p_15457 |  | GGGGCTTTTTTGTGTCCAGGAA |
| T. profu_5p_50983 | AMRN01000010.1_50983 | TATGGCGTTGCTGTAGTGTTTG |
| T. profu_3p_50983 |  | TAGTGTTTGCACTGGTGGTTAT |
| T. profu_5p_5000 | AMRN01000001.1_5000 | TGGGTAGGTGTTCACCTATCGG |
| T. profu_3p_5000 |  | GGTAACGAACTGTAATTACACT |
| T. profu_5p_480 | AMRN01000001.1_480 | CCCCCCGTCTGTCGTTCTTCTG |
| T. profu_3p_480 |  | TGGAAATCGCCAAGATGCGCGC |
| T. profu_5p_14005 | AMRN01000002.1_14005 | AGGGCCACGCCCAGGTCAATGC |
| T. profu_3p_14005 |  | CACATGGCCGGATGCAGCCAAC |
| T. profu_5p_44486 | AMRN01000008.1_44486 | GGACGCATTGATGCGACCCGAG |
| T. profu_3p_44486 |  | CCGAGTTCGGGCAGGTGTGCCA |
| T. profu_5p_1328 | AMRN01000001.1_1328 | TGATCCTCACAGTGTTGACCGC |
| T. profu_3p_1328 |  | AATCGCGCTTGGCTTTATTGGC |
| T. profu_5p_18148 | AMRN01000002.1_18148 | TGCCGCCGCGACCATCGCGGGG |
| T. profu_3p_18148 |  | CCCCGACGCTGATCGCGCGGGG |
| T. profu_5p_12680 | AMRN01000002.1_12680 | ATCGGGACAAATCGGTGCCGAT |
| T. profu_3p_12680 |  | ATGGTGGCATTGGCAACCGCAC |
| T. profu_5p_12074 | AMRN01000001.1_12074 | ATCGGGGCCGGATCCGGCGGCC |
| T. profu_3p_12074 |  | CCGGTGCGGTTCAGATGGGCGC |
| T. profu_5p_53216 | AMRN01000012.1_53216 | CTCTTTCCGCTGAACTAAGCT |
| T. profu_3p_53216 |  | CATGTTTCATCGGAGGGACATC |
| T. profu_5p_24324 | AMRN01000003.1_24324 | CGTCGGTCGTGAGCTGGCGCGC |
| T. profu_3p_24324 |  | TGGCGCGCTGGAGCCCGGAACA |
| ***T. xianhensis* P-4^T^** | | |
| T. xian_5p_16668 | CP004388.1_16668 | AGACGTGACCTTCGGGTCGCGT |
| T. xian_3p_16668 |  | CGTCTTTTTTATTGTCTGGTGG |
| T. xian_5p_6822 | CP004388.1_6822 | CAATTAAAAACCCCCTCAGGCG |
| T. xian_3p_6822 |  | AGGGGTTTTTTAATTGGTAGCC |
| T. xian_5p_20844 | CP004388.1_20844 | CGCAGCTTTCCGGCTGCGGGGC |
| T. xian_3p_20844 |  | GGGCTTTTTTACATTTGGTTGC |
| T. xian_5p_2740 | CP004388.1_2740 | GAGTCCATGGTGCTGCGAGCTC |
| T. xian_3p_2740 |  | ATTCCCGCGTGCGCGGGAATGA |
| T. xian_5p_9958 | CP004388.1_9958 | ATATTTCGTAACCATATTTACG |
| T. xian_3p_9958 |  | AAATATCGTAGGCCGATCACGA |
| T. xian_5p_3333 | CP004388.1_3333 | ATCATCGGTGACGGTGGTGGCT |
| T. xian_3p_3333 |  | AGCCATCGCTGCCGGTGATCCC |
| T. xian_5p_15747 | CP004388.1_15747 | CTTCTTTAACGAACTTGAAGAA |
| T. xian_3p_15747 |  | AGAATGGCTTTCGGGCTTTACC |
| T. xian_5p_5035 | CP004388.1_5035 | CCCGGGACATGTCCCGGGCGAA |
| T. xian_3p_5035 |  | GGCGAAGTTTTGTGTGCGTATC |
| T. xian_5p_21040 | CP004388.1_21040 | AAAACCTCTTGCGCGGTCTTGA |
| T. xian_3p_21040 |  | CGCGCAAGAGGCTTTTGCATCG |
| T. xian_5p_3489 | CP004388.1_3489 | ACGGATCGTTGGAAAACCGTCG |
| T. xian_3p_3489 |  | AAACCGTCGACCGGGAGCTCGG |
| T. xian_5p_22881 | CP004388.1_22881 | CACCATCCTGACCGCCAGCATG |
| T. xian_3p_22881 |  | TATTTATCGGGTTGGGCGGCGA |
| T. xian_5p_14589 | CP004388.1_14589 | TATCCTCAAAGCCGACGACCGA |
| T. xian_3p_14589 |  | ACTCCCGCTTTCGCCGGAGTGA |
| T. xian_5p_2738 | CP004388.1_2738 | GAGTCCACCGGGCTTGCGGGTT |
| T. xian_3p_2738 |  | ATTCCCGCGTGCGCGGGAATGA |
| T. xian_5p_12893 | CP004388.1_12893 | AATGGGCGGGGCAACCCGCGAT |
| T. xian_3p_12893 |  | GATCTTTTCCATTGGATATCGG |
| T. xian_5p_22216 | CP004388.1_22216 | CCCCGTACTTTGTACGGGGCTT |
| T. xian_3p_22216 |  | GGGCTTCTTTTTTTGCCGGTCG |
| T. xian_5p_17206 | CP004388.1_17206 | GGCGTCGGGCCTTGATGGCCGT |
| T. xian_3p_17206 |  | GCCGCAAGACCGCCGCCGCCAA |
| T. xian_5p_19488 | CP004388.1_19488 | TCGGTTTCTGCCTTGGCGGGCT |
| T. xian_3p_19488 |  | CACGCGAACTGATGGCGATGCC |
| T. xian_5p_3512 | CP004388.1_3512 | AATCAGCCTGATTATTCACTTT |
| T. xian_3p_3512 |  | ATTCACTTTTTGCATTGCGGCA |
| T. xian_5p_20129 | CP004388.1_20129 | AAGGGTCGCCCAGACATCGCCA |
| T. xian_3p_20129 |  | TTGCGGGTAAAACTGGCGATGC |
| T. xian_5p_6229 | CP004388.1_6229 | AGGGCATCGGTCAGGCCTTTGG |
| T. xian_3p_6229 |  | AATGCATGCATGGCAAAACCGA |
| T. xian_5p_4741 | CP004388.1_4741 | CTGCCGATTGTGCTGCTGATCC |
| T. xian_3p_4741 |  | GGGTGATCGTAGCACTCCGGCG |
| T. xian_5p_17016 | CP004388.1_17016 | AATGGCCAGCTTTCCCCGATCC |
| T. xian_3p_17016 |  | ATGCAAGGTTATTGATCGGACC |
| T. xian_5p_16719 | CP004388.1_16719 | ATTTTGGATGCCTTGCGTGTTT |
| T. xian_3p_16719 |  | GAAGGAATTCATCATTTTGCCA |
| T. xian_5p_22099 | CP004388.1_22099 | TTGCCGGCTGTGATCATCATGT |
| T. xian_3p_22099 |  | TGGTGTCGGGCCTGCGCGAACA |
| T. xian_5p_16407 | CP004388.1_16407 | CATTTTCCGCCTCGCAGACTTT |
| T. xian_3p_16407 |  | CCTGCGCGATTGCGCTATCGAT |
| T. xian_5p_18366 | CP004388.1_18366 | TATAATGCAACCGAAAGCCGTC |
| T. xian_3p_18366 |  | GGCGGCTTTCGCGTTTTGGGGG |
| T. xian_5p_4407 | CP004388.1_4407 | CAACAGTGCGGCCGAGGCGCGT |
| T. xian_3p_4407 |  | TGCGCAGCACGGTTGCCGATAT |
| T. xian_5p_17681 | CP004388.1_17681 | CTATTCCATCGGTTACATGTCG |
| T. xian_3p_17681 |  | CATGTCGCACGATCCCGACAAG |
| T. xian_5p_23642 | CP004388.1_23642 | TCTGGGCCTGCCGACGGTTCTT |
| T. xian_3p_23642 |  | CATTGTTCTCGTAGGCCCGCCA |
| T. xian_5p_3091 | CP004388.1_3091 | ATCGGCGGCAGTTTTGGCGTCC |
| T. xian_3p_3091 |  | CCAAAGATGCTGCCGTCGCCCA |
| T. xian_5p_22837 | CP004388.1_22837 | ATTGACCTTGGTTTCCAGCGTG |
| T. xian_3p_22837 |  | CAGCGTGTGATCGAAATCTGGT |
| T. xian_5p_17518 | CP004388.1_17518 | AGCTGTGCCGCGTCATGTTGCC |
| T. xian_3p_17518 |  | AACGGGCGCTGGGCAAAGTTGC |
| ***T. lucentensis* QMT2^T^** | | |
| T. luce_5p_167793 | ATWN01000002.1_ 167793 | TATCCACTAGACATCATTTGGA |
| T. luce_3p_167793 |  | TTGGATAATTATATAACAATTT |
| T. luce_5p_124614 | ATWN01000002.1 _124614 | TAAAGAACAAAAGCTCCGCAGG |
| T. luce_3p_124614 |  | GAGCTTTGTTTTTTGCGTAAAA |
| T. luce_5p_247382 | ATWN01000003.1_247382 | CCAACCGGGTGTTTAACCCGCA |
| T. luce_3p_247382 |  | AGTATGGGCAGGCTCAATCGCG |
| T. luce_5p_523350 | ATWN01000010.1_523350 | ATGTCCATGACCGTGGTGGTGA |
| T. luce_3p_523350 |  | TGACCATGACCATGACCATGAC |
| T. luce_5p_637434 | ATWN01000020.1_637434 | TTTTGTGGCACCTGACAACTTT |
| T. luce_3p_637434 |  | CGAGTGCCACAAAAGATGACAT |
| T. luce_5p_312757 | ATWN01000005.1_312757 | AACCAACCATTATCGGTAAACA |
| T. luce_3p_312757 |  | TTTTACCGTAATGGTTGGTTTT |
| T. luce_5p_608218 | ATWN01000013.1_608218 | TGGTCTTTTCAAACCAGCTCGA |
| T. luce_3p_608218 |  | TCGATGCCGGTGGGCTTGACCG |
| T. luce_5p_496507 | ATWN01000009.1_496507 | AGATTTTCAAAAAAGGCGGTGT |
| T. luce_3p_496507 |  | CACCGCTTTTTTTATATCCGGA |
| T. luce_5p_269288 | ATWN01000004.1_269288 | CATTGATGCATCAATGATACGT |
| T. luce_3p_269288 |  | TCAATGATGCATTCGAGTATCA |
| T. luce_5p_559608 | ATWN01000011.1_559608 | ATATGCGATGATCTATGGCTGC |
| T. luce_3p_559608 |  | TTTGGCGTTGCCTACGCCTATT |
| T. luce_5p_170616 | ATWN01000002.1_170616 | CCCGGCCCTTGCCTATGCTGGC |
| T. luce_3p_170616 |  | CTTTGCAATTGCCAAGAAGGAA |
| T. luce_5p_158773 | ATWN01000002.1_158773 | AAACTGCCAGCTCGTGCCGTTT |
| T. luce_3p_158773 |  | TTTCGGTCCGGATGATGCCGGA |
| T. luce_5p_123341 | ATWN01000002.1_12334 | CAGCCCAAGCCCGCGCTTGGGC |
| T. luce_3p_123341 |  | GGGCTGTTTTTCTTCAATCAGG |
| T. luce_5p_528656 | ATWN01000010.1_528656 | TTCGGTGCTCACGTACTTTTAG |
| T. luce_3p_528656 |  | TGCGCTCCGATGCGCGTGAACC |
| T. luce_5p_542101 | ATWN01000010.1_542101 | TGTCCTGTGGACACTCATATTG |
| T. luce_3p_542101 |  | AATATGAGTGTCCACAGGCTCT |
| T. luce_5p_39407 | ATWN01000001.1_39407 | CCGGAACGGTCCAGTTGTTCCG |
| T. luce_3p_39407 |  | CGGCCTTTCAGGGTGCCTTCGC |
| T. luce_5p_185415 | ATWN01000002.1_185415 | AAGTTTCTTCCCACCCTTTTGA |
| T. luce_3p_185415 |  | AGGAAGAAATATCCCGCCTAAC |
| T. luce_5p_248131 | ATWN01000003.1_248131 | ACGATTTTGTCGGCCATGGTCA |
| T. luce_3p_248131 |  | TGATCATGGGTGACATAGATCA |
| T. luce_5p_429076 | ATWN01000007.1_429076 | AAGCCCGCCGGTTTGGCGGGCT |
| T. luce_3p_429076 |  | CGGGCTTTTGTCTGGCAACATA |
| T. luce_5p_228121 | ATWN01000003.1_228121 | AATAAAAGACCCTGCCACCGTG |
| T. luce_3p_228121 |  | AGGGTCTTTTTATTGGTTGCGG |
| T. luce_5p_543369 | ATWN01000011.1_543369 | AAGGCCGCAGCATGATGCTGCG |
| T. luce_3p_543369 |  | TGCGGCCTTTGTCATTCACATC |
| T. luce_5p_302533 | ATWN01000004.1_302533 | CCGCCTTCGCGGGGATGACGGT |
| T. luce_3p_302533 |  | TCATTCCCGCGCAGGCGGGAAT |
| T. luce_5p_498549 | ATWN01000009.1_498549 | ATTCAAGCTGGCTGATCATTGG |
| T. luce_3p_498549 |  | CGTCAGTTTCAACCACATGCTT |
| T. luce_5p_48057 | ATWN01000001.1_48057 | CACGGATTTCTCTGTCGATATG |
| T. luce_3p_48057 |  | TGTCGATATGGAAACGCTTAAG |
| T. luce_5p_528636 | ATWN01000010.1_528636 | TTCGGTGCTCACGTACTTTTAG |
| T. luce_3p_528636 |  | TGCGCTCCGATGCGCGTGAACC |
| T. luce_5p_525349 | ATWN01000010.1_525349 | AAGAGCCTGTGGACACTCATAT |
| T. luce_3p_525349 |  | CAATATGAGTGTCCACAGGACA |
| T. luce_5p_285535 | ATWN01000004.1_285535 | TTTGAGGAATTCCTCGATCAGC |
| T. luce_3p_285535 |  | ATCAGCGGGAAATAATGAATTG |
| T. luce_5p_13919 | ATWN01000001.1_13919 | AAAGGACGAAGTGTCCGGTCCG |
| T. luce_3p_13919 |  | GGTCCGATTACCTTACCGCCAT |
| T. luce_5p_389406 | ATWN01000006.1_389406 | TCGTGCGTCAGCTTGGCGTGAC |
| T. luce_3p_389406 |  | TCACCCGACCTGACCATGGTCG |
| T. luce_5p_307016 | ATWN01000004.1_307016 | AACTTGCCGGGGCTAATACGGT |
| T. luce_3p_307016 |  | AATTACCGTATCCGCCCCGGCG |
| T. luce_5p_471094 | ATWN01000008.1_471094 | TTGCCAAATTCAAAAGGCGGTG |
| T. luce_3p_471094 |  | CGCCGCCTTTTTTGTGTGCATA |
| T. luce_5p_311628 | ATWN01000005.1_311628 | ACCCGCATGAAATGCCGGTCGT |
| T. luce_3p_311628 |  | CCGGTCGTCTTCTGTAAACGCG |
| T. luce_5p_384973 | ATWN01000006.1_384973 | TCATCGCGCACAAAGCCATTGT |
| T. luce_3p_384973 |  | TGTCGTTTTGTGCCGATAACGA |
| T. luce_5p_7447 | ATWN01000001.1_7447 | GCGGTGGCATGGTCACCAACGC |
| T. luce_3p_7447 |  | AACGCATCAGAAAGGCGTTGAT |
| T. luce_5p_245911 | ATWN01000003.1_245911 | TGGGGCCAAATGTGATCTTGGT |
| T. luce_3p_245911 |  | CATGGGCGCGTTTGGCCTGTGC |
| T. luce_5p_879 | ATWN01000001.1_879 | ATGCCAAAAGAAAAACACCCCG |
| T. luce_3p_879 |  | AGCGGGGTGTTTTGTTAAGCTT |
| T. luce_5p_464921 | ATWN01000008.1_464921 | TGTGGCCGTTTTTGGTCATCAC |
| T. luce_3p_464921 |  | GATGACAATTACGGTCGGGATT |
| T. luce_5p_171055 | ATWN01000002.1_171055 | CCAGCTTCCTGCCGCCAACAGA |
| T. luce_3p_171055 |  | AACAGATCAAGTGACGGTGCTT |
| T. luce_5p_198770 | ATWN01000003.1_198770 | TGACTGGCGAATATGACGTCAT |
| T. luce_3p_198770 |  | CATGTTGCCGTTTCGCGATGGT |
| T. luce_5p_350700 | ATWN01000005.1_350700 | ACGTGACATGATGCCGGGCCGA |
| T. luce_3p_350700 |  | TTCGGCCCGGTTTGCATTTGTT |
| T. luce_5p_82704 | ATWN01000001.1_82704 | AATATCGCAAGGGCGTTCTGGG |
| T. luce_3p_82704 |  | TGCCATCGAAGCCCTTTCGGTG |
| T. luce_5p_273434 | ATWN01000004.1_273434 | CAGGTCAAAAAAAGCGCAACGG |
| T. luce_3p_273434 |  | GGTTGCGCTAGTTTTTGGCCGT |
| T. luce_5p_273440 | ATWN01000004.1_273440 | AAATCAAAATTACTTAATTTTA |
| T. luce_3p_273440 |  | TTTATTTTCGATTATTTTTATG |
| T. luce_5p_142582 | ATWN01000002.1_142582 | GCTCTGCAAGACGTTGCTGACA |
| T. luce_3p_142582 |  | TGACATGAATGGTGGCCCGGTG |
| T. luce_5p_449960 | ATWN01000008.1_449960 | TATGGGCTTTATCGCCTGCACA |
| T. luce_3p_449960 |  | AAATGTTTGTCGCCTTTGCCCG |
| T. luce_5p_437928 | ATWN01000007.1_437928 | TAAAAAAGGGCGGCCTGTCATA |
| T. luce_3p_437928 |  | AGGTCGCCCTTTTTCGTTAACT |
| T. luce_5p_127263 | ATWN01000002.1_127263 | TGTCGGGTTGAACGACAGCGCA |
| T. luce_3p_127263 |  | CGCACGATGCCATTGGAAGCGG |
| T. luce_5p_210055 | ATWN01000003.1_210055 | TCGGCGGATTTTTGACCCGATT |
| T. luce_3p_210055 |  | ATTTAAAGTCCGGTTCGACCGC |
| T. luce_5p_432525 | ATWN01000007.1_432525 | CGCGCATTCCTCAATGCGGTGT |
| T. luce_3p_432525 |  | TGCGGTGTCAAACGGGGTGCTG |
| T. luce_5p_112640 | ATWN01000002.1_112640 | ATCGGCACCAATTTTGCCCTTT |
| T. luce_3p_112640 |  | CGGCAAAACTGGTGCCGATTAA |
| T. luce_5p_529459 | ATWN01000010.1_529459 | CGGTGCAAAAGTCGCCGCTATA |
| T. luce_3p_529459 |  | CGCTATAGCTGCTTTGCCAGTT |
| T. luce_5p_48943 | ATWN01000001.1_48943 | TTTGCGGCGTCCTTTCAATCGT |
| T. luce_3p_48943 |  | CGCCGCCTTTTTCGTGGTTGTC |
| T. luce_5p_294505 | ATWN01000004.1_294505 | AACGGCCTATTTGGGATGAAGC |
| T. luce_3p_294505 |  | GGTCTCAAACGGCCCGGCAATT |
| T. luce_5p_485577 | ATWN01000009.1_485577 | CCCGGTCTGGAATCGGTTGGCG |
| T. luce_3p_485577 |  | GGTTGGCGATCGTACGTATGTG |
| T. luce_5p_475755 | ATWN01000008.1_475755 | AGCTTGGCAGGAATGAATGGTG |
| T. luce_3p_475755 |  | CCACTCATTTTCTTGCAAAGAT |
| T. luce_5p_601689 | ATWN01000013.1_601689 | CGCCCCGCAGATTTGCGGGGCG |
| T. luce_3p_601689 |  | GGGGCGTTTTTTTGTGCCGGTC |
| T. luce_5p_385818 | ATWN01000006.1_385818 | CAAAGGATTGCTTGGCCGCCCC |
| T. luce_3p_385818 |  | TTGGCCGCCCCGAAAGCTACCT |
| T. luce_5p_338996 | ATWN01000005.1_338996 | TGATCCAGACCGGCGAGGTCGG |
| T. luce_3p_338996 |  | CCTGTTACTCGCCGGTCTTTTT |
| T. luce_5p_17121 | ATWN01000001.1_17121 | AGTGCTGCATCGCTCAGGCGCA |
| T. luce_3p_17121 |  | TGCGCGACGTTGCAGCACAAGT |
| T. luce_5p_56287 | ATWN01000001.1_56287 | TTGCGGTTCTGTCGAACTCGCC |
| T. luce_3p_56287 |  | AACTCGCCAGAACGGTTTTTAA |
| T. luce_5p_218949 | ATWN01000003.1_218949 | ATCAGGTCGAAGCCATGACCAT |
| T. luce_3p_218949 |  | ACCATGGCCGACAAAATCGTCG |
| T. luce_5p_314181 | ATWN01000005.1_314181 | TGGATGTTATGGATGGGCGGCG |
| T. luce_3p_314181 |  | CATCTATCTGAAGTTAAGTAAG |
| T. luce_5p_441886 | ATWN01000007.1_441886 | AATATGGATCGGCAGTGGTTCG |
| T. luce_3p_441886 |  | TTAGAACCACTTCAAGCAATCC |
| T. luce_5p_441217 | ATWN01000007.1_441217 | TTTACACCGGCAAGGTCGGCTT |
| T. luce_3p_441217 |  | AACGCTGGGTGGTTGTCGCCCC |
| T. luce_5p_156594 | ATWN01000002.1_156594 | GTTATTGCAGGAATAGGCAATG |
| T. luce_3p_156594 |  | AATGCATGATCCTGTGTAACGA |
| T. luce_5p_260951 | ATWN01000004.1_260951 | ATTGCTTTACAGCAGCCCGATC |
| T. luce_3p_260951 |  | CCCGATCATCTGCTTGGGGCGA |
| T. luce_5p_641508 | ATWN01000022.1_641508 | AAGCAAAAAGACTGGGGCCGGA |
| T. luce_3p_641508 |  | ATCCGCCCACGGGCTTGTTGCC |
| T. luce_5p_109433 | ATWN01000001.1_109433 | ACTTGCCACATCCTGACGGCCC |
| T. luce_3p_109433 |  | AGTACGGTAAATTCACCGTGCT |
| T. luce_5p_556182 | ATWN01000011.1_556182 | TCGGGTCAGGAGCGGATCGGGG |
| T. luce_3p_556182 |  | GGGATTTTGGCCCGGCATCGCC |
| T. luce_5p_399471 | ATWN01000006.1_399471 | CAACTGTCATCGCGTATCCGGT |
| T. luce_3p_399471 |  | TTGCTGGTTGATTTTATGCATG |
| T. luce_5p_492342 | ATWN01000009.1_492342 | ACCTTACGGTTTAAGCAGGCGT |
| T. luce_3p_492342 |  | AAGCAGGCGTTTGAAATGTCAT |
| T. luce_5p_413359 | ATWN01000007.1_413359 | ATCGGTGTTGCAACTGCCGCCG |
| T. luce_3p_413359 |  | CGCCGCCGGGATCATTGTGGGC |
| T. luce_5p_520914 | ATWN01000010.1_520914 | TTAATCCGGACCCATTAATTAT |
| T. luce_3p_520914 |  | CATAATTAATGTGTTCGGAACT |
| T. luce_5p_23446 | ATWN01000001.1_23446 | CCAGTGTGTGGGGCGGCAGTTC |
| T. luce_3p_23446 |  | CAGTTCAGATGAGGAAAGGTGG |
| T. luce_5p_609984 | ATWN01000014.1_609984 | GTGATGATTTTTTCTTCACCGC |
| T. luce_3p_609984 |  | AGGTCGATCGCAACGCGTGCTT |
| T. luce_5p_608572 | ATWN01000013.1_608572 | ATCGCGTCCCAACGCCGGACGC |
| T. luce_3p_608572 |  | CGCATTTCAAGGGCCAAGGGCA |
| T. luce_5p_558336 | ATWN01000011.1_558336 | TTGATGACGATATTGACGTTCA |
| T. luce_3p_558336 |  | AATTTAGTCTTTCGACGATCAG |
| T. luce_5p_160251 | ATWN01000002.1_160251 | TAATGACAGCATTACCATCATC |
| T. luce_3p_160251 |  | CATCATCGTCAATGCCGATCTT |
| T. luce_5p_31255 | ATWN01000001.1_31255 | TTTCCTGACTTTTGGCGGTCGA |
| T. luce_3p_31255 |  | GGTTTGCCAATGGGCAGAAACC |
| T. luce_5p_43818 | ATWN01000001.1_43818 | AGAATTATCTATTGCGCAAGTG |
| T. luce_3p_43818 |  | TGCGCAAGTGCGAGATAATTGC |
| T. luce_5p_66984 | ATWN01000001.1_66984 | CTCGGCCTGCGCCTTGCGGCCG |
| T. luce_3p_66984 |  | CCGAGGTTTTCGCGGATCGCGA |
| T. luce_5p_678 | ATWN01000001.1_678 | ATCATGTTTTCAAGGGTTTCCG |
| T. luce_3p_678 |  | CAGTACATGTTCGGTATCGATG |
| T. luce_5p_533917 | ATWN01000010.1_533917 | CCCCGCTATATGCGGGGCTGAG |
| T. luce_3p_533917 |  | GATTTTGCGTGCCCGCGCACGC |
| T. luce_5p_262588 | ATWN01000004.1_262588 | CCGGCACAGTGTTTGCTGGATG |
| T. luce_3p_262588 |  | CTGGATGGAAAGTCAGAAATCC |
| T. luce_5p_246051 | ATWN01000003.1_246051 | ATCAGGTCATGCAATTTGGCGA |
| T. luce_3p_246051 |  | ACTGTCGCACCATCGTTTTGAC |
| T. luce_5p_65195 | ATWN01000001.1_65195 | TTCCTTCTGGCATGGAGCGGAA |
| T. luce_5p_65195 |  | AGCGGAACCGTAGAACCGGCAA |
| T. luce_5p_531055 | ATWN01000010.1_531055 | TTGCGCTATCGATTCGCAACTG |
| T. luce_3p_531055 |  | AACTGGCGGGCGCGGGTGAGGA |
| T. luce_5p_465632 | ATWN01000008.1_465632 | AATCGGAAGCGACAAACGGCGC |
| T. luce_3p_465632 |  | AACGGCGCGTCCCAAGGGGCGC |
| T. luce_5p_458430 | ATWN01000008.1_458430 | CACGTGGCTTTGATGGTGCGGC |
| T. luce_3p_458430 |  | ATGAACTATCAGTTCCACGACA |
| T. luce_5p_22792 | ATWN01000001.1_22792 | CGATTGTGTTCTGCTTGCGTCG |
| T. luce_3p_22792 |  | TGCGTCGTCATGTGATGCGGTT |
| T. luce_5p_282136 | ATWN01000004.1_282136 | TGCCGGTATTTCGGCCGATGAC |
| T. luce_3p_282136 |  | GATGACCTGAAGGTCTTCAAAT |
| T. luce_5p_43186 | ATWN01000001.1_43186 | CCGTGAAGTAGGCATTCAGAAT |
| T. luce_3p_43186 |  | CGGGTCTGCTTTCTCACTCATG |
| T. luce_5p_637355 | ATWN01000019.1_637355 | GGTTTAATACCGGAATCTGATG |
| T. luce_3p_637355 |  | CGGAATCTGATGCCGGTTTAAT |
| T. luce_5p_273060 | ATWN01000004.1_273060 | GGGTGGCGATTTTGGCCGGTGT |
| T. luce_3p_273060 |  | AATTGCCAAGCTCGGCCACCTG |
| T. luce_5p_21030 | ATWN01000001.1_21030 | TGTGCGAGGCAACACGTTGCGA |
| T. luce_3p_21030 |  | CGTTGCGATGAACAAAGCTTGA |
| T. luce_5p_617474 | ATWN01000014.1_617474 | TGATTACAACGGCTGCACGGCA |
| T. luce_3p_617474 |  | AAGTTGATTTCGAAATTGATTT |
| T. luce_5p_394944 | ATWN01000006.1_394944 | CCCAGCATGATCCGTTCGGCGG |
| T. luce_3p_394944 |  | GGTGACGGTTGTTGTTGATCCG |
| T. luce_5p_284817 | ATWN01000004.1_284817 | GGCACGCTTTACATGTGCCTGG |
| T. luce_3p_284817 |  | TTGATCTGCGCGCGGCCCTTCG |
| T. luce_5p_252989 | ATWN01000003.1_252989 | AGATTGATCTCGACGATGCGTT |
| T. luce_3p_252989 |  | CGATGCGTTCGACACCGCCAGC |
| T. luce_5p_252989 | ATWN01000003.1_252989 | TTGTTACTGACCAGAAAGAGGG |
| T. luce_3p_252989 |  | AAGAGGGCGGTGTCACCGTTGT |
| T. luce_5p_38961 | ATWN01000001.1_38961 | CAGACCATTTTGGTTCTGCCAG |
| T. luce_3p_38961 |  | AATCGGCGGTATCACCGTTATC |
| T. luce_5p_585162 | ATWN01000012.1_585162 | GTGATGATCAGCATAGTATCAT |
| T. luce_3p_585162 |  | ATCATCATACAATAGGATAAGT |
| T. luce_5p_389408 | ATWN01000006.1_389408 | TCGTGCGTCAGCTTGGCGTGAC |
| T. luce_3p_389408 |  | TCACCCGACCTGACCATGGTCG |
| T. luce_5p_474240 | ATWN01000008.1_474240 | GAAGTTAAGTGAAACCCCCATC |
| T. luce_3p_474240 |  | TGGGGGTTTTGCTTTAGGGGCG |
| T. luce_5p_421879 | ATWN01000007.1_421879 | AAAGCCTAAAAAGAAACAAGCC |
| T. luce_3p_421879 |  | AACTGGCTTGTTTTTATAGGTT |
| T. luce_5p_442710 | ATWN01000007.1_442710 | CGGATCGGTCACCTGCGCCATA |
| T. luce_3p_442710 |  | CCATACGTGCACGGGTGTCGTC |
| T. luce_5p_346590 | ATWN01000005.1_346590 | CGGGCTTATTTCATGAGTCCGG |
| T. luce_3p_346590 |  | TCCGGCCCTTGTTTTTTTAGAT |
| T. luce_5p_544598 | ATWN01000011.1_544598 | AAAAGAAACCGCCGCCCGGCTT |
| T. luce_3p_544598 |  | TGGCGTTTTCTTTTGTGTCCTG |
| T. luce_5p_106175 | ATWN01000001.1_106175 | GAATAATCTGGCTGATTATTCA |
| T. luce_3p_106175 |  | ATTCACTTTTAGTTGCGATCCG |
| T. luce_5p_406605 | ATWN01000007.1_406605 | CGAAATTTCGATGATGTGGCGG |
| T. luce_3p_406605 |  | CAGTTCGACCGACATTTTGGCG |
| T. luce_5p_58702 | ATWN01000001.1_58702 | GTGATGTGTCTATTCAGGAATT |
| T. luce_3p_58702 |  | ACGTCCTGATTCAGTTGCAGCG |
| T. luce_5p_422620 | ATWN01000007.1_422620 | GACGGCCTTGATGAGGCCGACG |
| T. luce_3p_422620 |  | GACGATCCGGGCGACATCCTTG |
| T. luce_5p_5460 | ATWN01000001.1_5460 | CTTCGCAATCGAAATGCGTATC |
| T. luce_3p_5460 |  | TGCGTATCAACTGGTCGGCCTT |
| T. luce_5p_316125 | ATWN01000005.1_316125 | GATGTGGAGAACCTGTTGGTCG |
| T. luce_3p_316125 |  | CCCAGCTTTCCCTGTCCCCGGA |
| T. luce_5p_546117 | ATWN01000011.1_546117 | AACGCCTTTATCGGCGCACGTT |
| T. luce_3p_546117 |  | AAGGTGTTATCGTCGAAGAAGG |
| T. luce_5p_273558 | ATWN01000004.1_273558 | AATCGTGTACGATTTAAATTAA |
| T. luce_3p_273558 |  | AAATTAATCGGCTTTGAGTCGT |
| T. luce_5p_399890 | ATWN01000006.1_399890 | ATAAACCGGCCCAATCGGATGC |
| T. luce_3p_399890 |  | TGTGTTGGTTTGTGTGTGACCC |
| T. luce_5p_242930 | ATWN01000003.1_242930 | ATATGACGGAAATCGGGAAGTC |
| T. luce_3p_242930 |  | CCGATATATCCGTCATTTTGCG |
| T. luce_5p_286186 | ATWN01000004.1_286186 | TGCCGATGGCGGCGCGACGCGT |
| T. luce_3p_286186 |  | CGATGTCGCCGCCCGCCTTGGC |
| T. luce_5p_481971 | ATWN01000009.1_481971 | TAATGCATTTCGATGTCGTCTT |
| T. luce_3p_481971 |  | CATAGAAGAACACGACAAAGGC |
| T. luce_5p_585272 | ATWN01000012.1_585272 | TGAGTTCATCAAGTGCGCGGCG |
| T. luce_3p_585272 |  | CGCTTTCGCAGGCTTGATGAGC |
| T. luce_5p_502941 | ATWN01000009.1_502941 | TATGTCGATCCGGATCGTATCC |
| T. luce_3p_502941 |  | TATGGTCCGCGATATTGCCCGC |
| T. luce_5p_123988 | ATWN01000002.1_123988 | AACGGTCGATCAGGACGGCAAG |
| T. luce_3p_123988 |  | TTGTTCTGACCCTTTCGACCCG |
| T. luce_5p_252720 | ATWN01000003.1_252720 | TAAGGGCTGTGCGCTCCGCCGT |
| T. luce_3p_252720 |  | CGCCGTCAGGATGCCGCGGGCA |
| T. luce_5p_313656 | ATWN01000005.1_313656 | ATACGATGGTTTTCCGGGCCAA |
| T. luce_3p_313656 |  | ACCGGTAACCGTTCGGTTTTGG |
| T. luce_5p_602433 | ATWN01000013.1_602433 | AAACCGATGAGGTTCTTTCGAT |
| T. luce_3p_602433 |  | CTTTCGATTGTTGGCGGTGCGC |
| T. luce_5p_589692 | ATWN01000012.1_589692 | TGATTTCGCCGTCGGCAAGGAT |
| T. luce_3p_589692 |  | ATCCTGCCCGGTGATGGTGATC |
| T. luce_5p_572238 | ATWN01000012.1_572238 | ACACATCAAGCAAACCGGGGCG |
| T. luce_3p_572238 |  | CCCGGTTTTGTTTTGTCTTCGA |
| T. luce_5p_559495 | ATWN01000011.1_559495 | GCAGGGCGACATCAACCTTGTG |
| T. luce_3p_559495 |  | ACGGGTTCGCGCATTCCTATAA |
| T. luce_5p_27103 | ATWN01000001.1_27103 | GGCGCACTCACTCAAAAGGTTA |
| T. luce_3p_27103 |  | CTTCTGGCAGGGCGACTCGGCG |
| T. luce_5p_181596 | ATWN01000002.1_181596 | TGTCGCGCCCGGCGTGCCCGGC |
| T. luce_5p_181596 |  | ATGCCGGTCATGTTGGGCGAAA |
| T. luce_5p_44717 | ATWN01000001.1_44717 | GCCGGTAATCCGGCGGCTGTTG |
| T. luce_3p_44717 |  | ATGGTTGCCTGATGACGTGCTT |
| T. luce_5p_184304 | ATWN01000002.1_184304 | CCTGTTGTTACGCGACGCATTA |
| T. luce_3p_184304 |  | CGCGACGCATTAAAACCCTGTG |
| T. luce_5p_8020 | ATWN01000001.1_8020 | AAAAGCCCGGCAGCAGGACAAA |
| T. luce_3p_8020 |  | CGGCCGGGCTTTTGCTGTTTTG |
| T. luce_5p_612357 | ATWN01000014.1_612357 | AATGAAAAACCCCGCAGACTGC |
| T. luce_3p_612357 |  | GGGGTTTTTACGTTCTCTGGGG |
| T. luce_5p_599500 | ATWN01000013.1_599500 | ACCGCTGGGCGCGCATGGTGGT |
| T. luce_3p_599500 |  | ACAGGCCTGCCTTACGTTTTAA |
| T. luce_5p_284324 | ATWN01000004.1_284324 | TAAAACAGCCCGGTGCCGTCCA |
| T. luce_3p_284324 |  | CCGGGCTGTTTTGTATGTTGGG |
| T. luce_5p_23450 | ATWN01000001.1_23450 | ATGTGCCTGTTGGCGCAGATCG |
| T. luce_3p_23450 |  | CAGATCGTCGAGGATTGTATTG |
| T. luce_5p_246818 | ATWN01000003.1_246818 | ATGCGGGTATGGGAACGCGGTG |
| T. luce_3p_246818 |  | TGCCGCCGGTGTTGCTGCGGCA |
| T. luce_5p_186751 | ATWN01000002.1_186751 | TCAGGGTCAAAGATCGGATTGT |
| T. luce_3p_186751 |  | CAGATTTCTTTGCCGCTCTTGG |
| T. luce_5p_374487 | ATWN01000006.1_374487 | ATCGAGGCCGCCGATACCGATC |
| T. luce_3p_374487 |  | AGTTCGGCCTATATTGGCGGCT |
| T. luce_5p_235876 | ATWN01000003.1_235876 | AATATTTATGCCCGGCCTCGAT |
| T. luce_3p_235876 |  | CATGATCGGTGCCGACCATGAT |
| T. luce_5p_46557 | ATWN01000001.1_46557 | CGCATGATTGATGAAGTCGGAT |
| T. luce_3p_46557 |  | TTCGGCGCATTAATCATATCGT |
| T. luce_5p_306227 | ATWN01000004.1_306227 | TGCGGGCGGGGTTGGCCCCGTC |
| T. luce_3p_306227 |  | TCGCAATTTCGGCAGTGCCCTG |
| T. luce_5p_234594 | ATWN01000003.1_234594 | TCATCGGACGGCCTGACTAAAC |
| T. luce_3p_234594 |  | TAAACCAATAAGATGGACGATT |
| T. luce_5p_522493 | ATWN01000010.1_522493 | CGTGCCTTATACTGCACGCTCC |
| T. luce_3p_522493 |  | ACGCTCCCTGCTTCGACACTTA |
| T. luce_5p_249012 | ATWN01000003.1_249012 | TGTGTGAACGAAACCGGCCTGC |
| T. luce_3p_249012 |  | TGCAGGCCGGTTTGCGTTTGCA |
| T. luce_5p_219290 | ATWN01000003.1_219290 | ACAGTTGCGCTGTTTTTATGGG |
| T. luce_3p_219290 |  | TTTTATGGGGGCGCTGGCGTCA |
| T. luce_5p_132929 | ATWN01000002.1_132929 | ATGTTGGCTTTGCCGTTGCTGG |
| T. luce_3p_132929 |  | TTGCCGTTGCTGGTCCCCGAAG |
| T. luce_5p_37686 | ATWN01000001.1_37686 | CGTATATGTCACACGGCATGTG |
| T. luce_3p_37686 |  | CGTGTGCGGCAAAGGTGATTGC |
| T. luce_5p_207159 | ATWN01000003.1_207159 | CTGGAGTTCGTGCATCGTGGGC |
| T. luce_3p_207159 |  | TGCGAAGCCGAAGTCCCGCCTC |
| T. luce_5p_258198 | ATWN01000004.1_258198 | TGAGGTGTATTTCAACCCGATG |
| T. luce_3p_258198 |  | TGACCATGTTGAACTCTTCCAT |
| T. luce_5p_345542 | ATWN01000005.1_345542 | CGCGGTCAGCCAAACCGGTTGA |
| T. luce_3p_345542 |  | TTGTTCTGACTGCACCGCTGGT |
| T. luce_5p_549646 | ATWN01000011.1_549646 | ACAGGTCATCCACGCCTGTTTC |
| T. luce_3p_549646 |  | TTTCGCTCGGTCTATGTCTGGT |
| T. luce_5p_394093 | ATWN01000006.1_394093 | TGACACTTGGCGATGGCAAAGG |
| T. luce_3p_394093 |  | TCGCCTTTGCTAAAATTTCATC |
| T. luce_5p_354019 | ATWN01000005.1_354019 | ATCTTCAGGCCGGTGAGATTGT |
| T. luce_3p_354019 |  | GCCGGTTTGCTGGGGTCGGGGC |
| T. luce_5p_593263 | ATWN01000013.1_593263 | AGGCTGCTGCTTCGCTCCGGGC |
| T. luce_3p_593263 |  | CCGCGTAAGCTCAACCTCGTCG |
| T. luce_5p_447654 | ATWN01000008.1_447654 | ATAAAATCTTCCTTCTGTTTGC |
| T. luce_3p_447654 |  | TGCCGGTCAGGCCGTTAAAATC |
| T. luce_5p_498538 | ATWN01000009.1_498538 | TCTGGCATCGGCGTTTCTATCG |
| T. luce_3p_498538 |  | CGTCGTCTGATCCGCTTTGCCA |
| T. luce_5p_275552 | ATWN01000004.1_275552 | TCGGGCAGGGGTTGATCGGCGG |
| T. luce_3p_275552 |  | TCGATACCGGGATATACGCCTG |
| ***T. australica* NP 3b2^T^** | | |
| T. aust_5p_9244 | JRJE01000004.1_scaffold_6_9244 | CAATTAAAAACCCCGCCAGGCT |
| T. aust_3p_9244 |  | CGGGGTTTTTAATTGGTAGCCG |
| T. aust_5p_18680 | JRJE01000006.1_scaffold_4_18680 | CGCCATTTTCAGGCCGAAGGCA |
| T. aust_3p_18680 |  | CCGAAGGCAATGTTGTTATAGA |
| T. aust_5p_16396 | JRJE01000005.1_scaffold_5_16396 | AGGAAAACCTCAAAGCGGTTTG |
| T. aust_3p_16396 |  | AAAGCGGTTTGTCTTGGCTGGG |
| T. aust_5p_48003 | JRJE01000031.1_scaffold_1_48003 | AACAGACCATCCAAGAGTCAGG |
| T. aust_3p_48003 |  | AGGTGAATGGAATTGCTGTTGA |
| T. aust_5p_52437 | JRJE01000031.1_scaffold_1_52437 | CTCGTTGGTGCACCAAACGCGG |
| T. aust_3p_52437 |  | AAACGCGGGCAAGTCAACGTTG |
| T. aust_5p_49513 | JRJE01000031.1_scaffold_1_49513 | CTCGATATTGTTTTGTCATTGA |
| T. aust_3p_49513 |  | CAATTTAAACATTGTCACCATG |
| T. aust_5p_4594 | JRJE01000003.1_scaffold_7_4594 | TTGCCTGCAGCCCTGCCGGCCT |
| T. aust_3p_4594 |  | CCTTTGCATCGGGCTTTCGTGT |
| T. aust_5p_11614 | JRJE01000004.1_scaffold_6_11614 | CCAGAGACCGAAGTCTCTGACA |
| T. aust_3p_11614 |  | GACAGTTCCAGCGTTATTAGCC |
| T. aust_5p_6624 | JRJE01000003.1_scaffold_7_6624 | CGGAATGAACAACGCCGGTGGG |
| T. aust_3p_6624 |  | ACCGGCGTTGTTGTTTCAAGGC |
| T. aust_5p_59292 | JRJE01000032.1_scaffold_0_59292 | TAATCTGCCCAAAGCTTGTCTT |
| T. aust_3p_59292 |  | GTCTTGGCTATCATGAAGTTCA |
| T. aust_5p_39387 | JRJE01000028.1_scaffold_12_39387 | GTCATTTGGCCTGTGCCGGGCG |
| T. aust_3p_39387 |  | TGCCGGGCGTTCCGCACTTGCA |
| T. aust_5p_42495 | JRJE01000030.1_scaffold_10_42495 | TTCATGGTCACCATGCTCGTCA |
| T. aust_3p_42495 |  | CATGATCATCGCCAGCATGGTC |
| T. aust_5p_4053 | JRJE01000002.1_scaffold_8_4053 | AATTGCGGTATTGTTGCCATCC |
| T. aust_3p_4053 |  | TTGCCATCCCGATTGCAATTGA |
| T. aust_5p_22590 | JRJE01000008.1_scaffold_30_22590 | ATGGTTGCCAAGATCATCGAAA |
| T. aust_3p_22590 |  | ACGCGATCCTGCCGACCATGGG |
| T. aust_5p_63319 | JRJE01000032.1_scaffold_0_63319 | CGATGCTTACGCCGGACCCATT |
| T. aust_3p_63319 |  | AAGCCTCATGATTAATGTGTTC |
| T. aust_5p_36180 | JRJE01000023.1_scaffold_17_36180 | CTGATTAGGAGTTGGCCGGGTT |
| T. aust_3p_36180 |  | AACCCAGTTTAATCTTAGTCAT |
| T. aust_5p_17558 | JRJE01000005.1_scaffold_5_17558 | CAGATGAATGGTAAAAGGCCGT |
| T. aust_3p_17558 |  | TGCGGCCTTTTATGTTTCAGGA |
| T. aust_5p_15365 | JRJE01000005.1_scaffold_5_15365 | AACGGGCGGTCGTAATGACCGC |
| T. aust_3p_15365 |  | CGCCCGTTTTTCGTTTCACCAG |
| T. aust_5p_5875 | JRJE01000003.1_scaffold_7_5875 | CAAAACGAAAACAGCGCCGGAC |
| T. aust_3p_5875 |  | CGGCGCTGTTTTCGTTTGTCGG |
| T. aust_5p_54777 | JRJE01000032.1_scaffold_0_54777 | CCCCGACAGAACATTCTGTCGG |
| T. aust_3p_54777 |  | CGGGGCTGTTTTTTTATCTGTG |
| T. aust_5p_5027 | JRJE01000003.1_scaffold_7_5027 | CGATTATTCGCTGGCGCTTTTT |
| T. aust_3p_5027 |  | CGCTTTTTTGTTTTCCGCCTCT |
| T. aust_5p_63384 | JRJE01000032.1_scaffold_0_63384 | TGAGCCTGGCGATTGGACGTCG |
| T. aust_3p_63384 |  | CCGCCTGGCATCCCAGCTGACC |
| T. aust_5p_3291 | JRJE01000002.1_scaffold_8_3291 | TGAATGGCAGACGGTATATTTG |
| T. aust_3p_3291 |  | AGTTTGTAAATATAACGTCTGT |
| T. aust_5p_56071 | JRJE01000032.1_scaffold_0_56071 | AGTGCCCGGTTTTTGCCGGGCA |
| T. aust_3p_56071 |  | CGGGCATTTTTGTTTGTGTTTG |
| T. aust_5p_2750 | JRJE01000002.1_scaffold_8_2750 | ATTGCGTGCAAGCATCGCGATT |
| T. aust_3p_2750 |  | CCCCCATTAACGGCATGAACGA |
| T. aust_5p_52364 | JRJE01000031.1_scaffold_1_52364 | TTGCTGCATCGACCGGCATTGT |
| T. aust_3p_52364 |  | TTGCTCAGCCTGCCGGCGATGC |
| T. aust_5p_61940 | JRJE01000032.1_scaffold_0_61940 | CCCGCCAGTTGCCTGGCGGGGG |
| T. aust_3p_61940 |  | CGGGGGTTTTGTTTCAGATCCG |
| T. aust_5p_56497 | JRJE01000032.1_scaffold_0_56497 | CACGGGTTTAATTGGCGAAGCT |
| T. aust_3p_56497 |  | CTTCGCCACTTTTCATTGGGAT |
| T. aust_5p_39756 | JRJE01000028.1_scaffold_12_39756 | AGGTTAATCTGGACTTCAGCTC |
| T. aust_3p_39756 |  | ACTTCAGCTCCGAAGCCGACAT |
| T. aust_5p_58885 | JRJE01000032.1_scaffold_0_58885 | AAAAACCCCGCAAGGTCACCTT |
| T. aust_3p_58885 |  | GGGGTTTTTCTTTTAATTGATG |
| T. aust_5p_56957 | JRJE01000032.1_scaffold_0_56957 | TGATTCCATCCTGGCGGCCGTA |
| T. aust_3p_56957 |  | AGACCCAGCTTGGTGGCGGCAT |
| T. aust_5p_54156 | JRJE01000031.1_scaffold_1_54156 | TGCCTTTTGTAATGGCAATGCC |
| T. aust_3p_54156 |  | AATGCCCGAAGACGGTGCCATG |
| T. aust_5p_3299 | JRJE01000002.1_scaffold_8_3299 | ACACACACCTGATTTGGGTTTT |
| T. aust_3p_3299 |  | TTTGGCCGATGATCGTGCCGGG |
| T. aust_5p_23133 | JRJE01000008.1_scaffold_30_23133 | GTGGAGCTGTCATTCACGGATG |
| T. aust_3p_23133 |  | ACTGTAAGATGAAGATCATCCG |
| T. aust_5p_20162 | JRJE01000006.1_scaffold_4_20162 | AGCGGCTTGCGCGACGTGTTGG |
| T. aust_3p_20162 |  | TGGCATGACTTCTAGCCCTGGT |
| T. aust_5p_37611 | JRJE01000025.1_scaffold_15_37611 | TCACGGCCGGTCTGTTCCTTGA |
| T. aust_3p_37611 |  | CGTCATTGAACAGATCGGTGCG |
| T. aust_5p_57006 | JRJE01000032.1_scaffold_0_57006 | GTTTTGCTTGTCGGGCAACGTG |
| T. aust_3p_57006 |  | AGCGTTGCCCATCAGTCAGGAT |
| T. aust_5p_17068 | JRJE01000005.1_scaffold_5_17068 | AATATCAACAACCTTGCTGCCG |
| T. aust_3p_17068 |  | TTCGGTCTGCTCGGTCTTGAAG |
| T. aust_5p_17456 | JRJE01000005.1_scaffold_5_17456 | CTGGGCTTCTGGCTCGTTGGTT |
| T. aust_3p_17456 |  | TGGTTTCCAGGAAGGCGTCATA |
| T. aust_5p_64344 | JRJE01000032.1_scaffold_0_64344 | TATGTCGCTGTCCTGCTGCATG |
| T. aust_3p_64344 |  | CATGATATCGCCAAGGGACGCG |
| T. aust_5p_10528 | JRJE01000004.1_scaffold_6_10528 | AGCGATGGCGGCTTGTCGTCGC |
| T. aust_3p_10528 |  | CGCTGGCATGGCGGATGGCGCA |
| T. aust_5p_968 | JRJE01000001.1_968 | GGTCCGGATCATTGGGCTGTCG |
| T. aust_3p_968 |  | ATGCAGATTTCAACCCGAACCA |
| T. aust_5p_57940 | JRJE01000032.1_scaffold_0_57940 | AGGGAAGCTGGTTGAACAAGAC |
| T. aust_3p_57940 |  | TGAGCCAGCTAGTGTCCTTTTG |
| T. aust_5p_7801 | JRJE01000003.1_scaffold_7_7801 | TCGCGCGGGCCATGACTTCCGA |
| T. aust_3p_7801 |  | GACTTCCGATCAAAAAGTGCGT |
| T. aust_5p_9946 | JRJE01000004.1_scaffold_6_9946 | AAATACTCGTTTGTTCTTGCGG |
| T. aust_3p_9946 |  | CAATGGGTTCAACAGCAAACAT |
| T. aust_5p_30915 | JRJE01000020.1_scaffold_2_30915 | TTCTGATCGAAGCCACGGGCGC |
| T. aust_3p_30915 |  | CATCCGTGGCGTTTAACGGTTT |
| T. aust_5p_28718 | JRJE01000009.1_scaffold_3_28718 | TGGCATCGGCGGTCGCGGCATC |
| T. aust_3p_28718 |  | TGGCCGGTGCGCTTTTTGCTCA |
| T. aust_5p_25487 | JRJE01000009.1_scaffold_3_25487 | AGCTCAGCCTGGTAGAGCACTG |
| T. aust_3p_25487 |  | ACTGTCTTCGGGAGGCAGGGGT |
| T. aust_5p_35000 | JRJE01000020.1_scaffold_2_35000 | AACCCGACGCTGGAGATTTAGA |
| T. aust_3p_35000 |  | TTTTAAAATCTCAGCTGCGGGC |
| T. aust_5p_16166 | JRJE01000005.1_scaffold_5_16166 | ATGTATGCCCGTCAGGATTGGG |
| T. aust_3p_16166 |  | CTGGACGGGGTGTGCATGGAAG |
| T. aust_5p_37584 | JRJE01000025.1_scaffold_15_37584 | TCAATGCGGTGGCACCGACGGT |
| T. aust_3p_37584 |  | AACTCGCTGCCGCCGCATGGTC |
| T. aust_5p_45945 | JRJE01000031.1_scaffold_1_45945 | TTCTATTTCCTGCTTATTCGTC |
| T. aust_3p_45945 |  | AAAGCAGAAAGAGCATAAAGCA |
| T. aust_5p_10418 | JRJE01000004.1_scaffold_6_10418 | TTTGAGCAAAAGCCCATAAAGA |
| T. aust_3p_10418 |  | TAAAGAATCACGTTTTGTTACC |
| T. aust_5p_54394 | JRJE01000031.1_scaffold_1_54394 | TGAAGCTTTGGACGGGGATGTT |
| T. aust_3p_54394 |  | CGTTTGAATTTCTTGACGCGCG |
| T. aust_5p_47340 | JRJE01000031.1_scaffold_1_47340 | TGATGTTGTTGCCAAAAAGCTT |
| T. aust_3p_47340 |  | CGTCGCATCGGTGTCTTCGATG |
| T. aust_5p_53512 | JRJE01000031.1_scaffold_1_53512 | CAAGTTCCGCTATGGCGAGACT |
| T. aust_3p_53512 |  | GATCAGGTCGGTGTTGTTACCG |
| T. aust_5p_53204 | JRJE01000031.1_scaffold_1_53204 | TGATGGCGATACCATCAAGGGG |
| T. aust_3p_53294 |  | ATCAAGGGGCAATGGCGCTGTT |
| T. aust_5p_46507 | JRJE01000031.1_scaffold_1_46507 | CGAAATGACAAAAGCGCACCGA |
| T. aust_3p_46507 |  | GTGCGCTTTTTTCTTTTCCATC |
| T. aust_5p_9275 | JRJE01000004.1_scaffold_6_9275 | ATCGGCTTTGCTTTAAGCTCGT |
| T. aust_3p_9275 |  | TCGTTCATTTTAAAGGCTTCCT |
| T. aust_5p_59139 | JRJE01000032.1_scaffold_0_59139 | CGGGCCTTCATTCGGGCGGTGG |
| T. aust_3p_59139 |  | CACGAAGAAGTCCCCAGCCCGA |
| T. aust_5p_32539 | JRJE01000020.1_scaffold_2_32539 | AGGGCGAAGGCAGCGTTGCGGA |
| T. aust_3p_32539 |  | CAAGTTGCTTTTGCCAATGGCG |
| T. aust_5p_34002 | JRJE01000020.1_scaffold_2_34002 | AAAGCGCAGGAGAGAAAGGTCA |
| T. aust_3p_34002 |  | CTTCTCTCCTGCAGCTCGTGAT |
| T. aust_5p_11971 | JRJE01000004.1_scaffold_6_11971 | CAAAACCGGTACGACCAATGAT |
| T. aust_3p_11971 |  | TTTGTTGGATTTTCGCCGGATT |
| T. aust_5p_23871 | JRJE01000008.1_scaffold_30_23871 | AACGTTCATCGTTTGGAACGTT |
| T. aust_3p_23871 |  | TGTGATATATCCGCGCAAATCC |
| T. aust_5p_51829 | JRJE01000031.1_scaffold_1_51829 | CAAGGGCGTTACTGAACGGCTT |
| T. aust_3p_51829 |  | CTTGGCCTGCAAATCAAGGCCG |
| T. aust_5p_4525 | JRJE01000002.1_scaffold_8_4525 | TTGATAATGCGGAATTCTCGGA |
| T. aust_3p_4525 |  | ATCTGGTCGCTGTTTTCAATAA |
| T. aust_5p_28537 | JRJE01000009.1_scaffold_3_28537 | TGGTGTCGATAAAAAAGGGGAA |
| T. aust_3p_28537 |  | TTCCCCTTTTTTGCGCGGAGAT |
| T. aust_5p_61509 | JRJE01000032.1_scaffold_0_61509 | ATATTTCCAACCCGCATGCGCG |
| T. aust_3p_61509 |  | CGCCATGTCAGTTGGATCAGTT |
| T. aust_5p_42691 | JRJE01000030.1_scaffold_10_42691 | TTTTCCTACTGGGAACATGAGA |
| T. aust_3p_42691 |  | ATGAGAACACAGTTGATATCTA |
| T. aust_5p_36995 | JRJE01000024.1_scaffold_16_36995 | ATCAGCCCATATCGTTTGATCG |
| T. aust_3p_36995 |  | AATTGCCCGTTCGACATTGTCG |
| T. aust_5p_16541 | JRJE01000005.1_scaffold_5_16541 | TGACCCTTGTGGTTTTCACCGG |
| T. aust_3p_16541 |  | CTGATTGCCCCGGGTTCCATTC |
| T. aust_5p_9684 | JRJE01000004.1_scaffold_6_9684 | AGCTGTTTCGCTTGAAACTTTG |
| T. aust_3p_9648 |  | CTTTGGATTTGTCGGCAAACGC |
| T. aust_5p_33837 | JRJE01000020.1_scaffold_2_33837 | ATTTGTCCGTCTGCTGCGGTGG |
| T. aust_3p_33837 |  | TTTTGGGCAAGGATGCGGTTGA |
| T. aust_5p_22981 | JRJE01000008.1_scaffold_30_22981 | AGTTGAAGCTGCGCCGGCCAGC |
| T. aust_3p_22981 |  | TGACCGACGCGGCTTCTTACTC |
| T. aust_5p_23424 | JRJE01000008.1_scaffold_30_23424 | TTTGGCGATTACCCGGGCCTTT |
| T. aust_3p_23424 |  | AGACCCTGGGTATTGCCATTCG |
| T. aust_5p_7549 | JRJE01000003.1_scaffold_7_7549 | CTTGGCATCACCGATGGCAAGA |
| T. aust_3p_7549 |  | TGGCAAGATTGCCGCCATCGCG |
| T. aust_5p_40281 | JRJE01000029.1_scaffold_11_40281 | CTGCGTCTCCTGCAGTAATCCG |
| T. aust_3p_40281 |  | AATCCGGATATCCGTAATGAGA |
| ***T. tepidiphila* 1-1B^T^** | | |
| T. tepi_5p_37581 | AMRN01000014.1_37581 | TTGATCGGGGATCACCCCGATC |
| T. tepi_3p_37581 |  | TCCTAGCTTTTTCTCTCATATC |
| T. tepi_5p_11218 | AMRN01000002.1_11218 | CTGTCGGAGACAGGGCATCTTG |
| T. tepi_3p_11218 |  | CCTGCCTCCGAACAGTCTGGGA |
| T. tepi_5p_7722 | AMRN01000001.1_7722 | AGCGACATCATCGGCACTTGCG |
| T. tepi_3p_7722 |  | TGTCGTTGATGTTGCTGCTAAT |
| T. tepi_5p_33812 | AMRN01000010.1_33812 | AAAGAAAAACCCCGCCAGATTG |
| T. tepi_3p_33812 |  | GGGGTTTTTTGTTGGTTTTGCC |
| T. tepi_5p_22906 | AMRN01000006.1_22906 | CATTATTATTGTGTCAATTTAA |
| T. tepi_3p_22906 |  | CAATTTAAACATTGTCACCATG |
| T. tepi_5p_9218 | AMRN01000002.1_9218 | CAAATCCCTTGACCGGGGATCA |
| T. tepi_3p_9218 |  | TTCCTAGTTTTTTCTCTCATAT |
| T. tepi_5p_33714 | AMRN01000010.1_33714 | AGATTGATCTGACGCGCGTTGA |
| T. tepi_3p_33714 |  | GTTGATGAAATTCGCCGGATGG |
| T. tepi_5p_24769 | AMRN01000006.1_24769 | AGCGACAACGCCGGTGGGATCA |
| T. tepi_3p_24769 |  | TGCCACCGGCGTTGTTGTCTTC |
| T. tepi_5p_6580 | AMRN01000001.1_6580 | TCTACTTTGTCGCAAGGCATTG |
| T. tepi_3p_6580 |  | CCTGCTGCCTTGCTTCGCGTAT |
| T. tepi_5p_9431 | AMRN01000002.1_9431 | AAGAAGCAGCGTCGGCCAGCCA |
| T. tepi_3p_9431 |  | GCCGGCGCTGCCTCAACTCGTT |
| T. tepi_5p_9402 | AMRN01000002.1_9402 | CCGTGTACTCGACCGCTTCATC |
| T. tepi_3p_9402 |  | GCGGTGCCATGGAGATCTGATG |
| T. tepi_5p_19158 | AMRN01000004.1_19158 | TGCAGGGTTCCTGCGCTGCCTT |
| T. tepi_3p_19158 |  | CGCTGCCTTTTTTGTTGCGATT |
| T. tepi_5p_2570 | AMRN01000001.1_2570 | TACGCCGGACCCATTACTCATG |
| T. tepi_3p_2570 |  | CTCATGAGTAATGTGTTCGGAA |
| T. tepi_5p_27975 | AMRN01000007.1_27975 | AATTTTCAGCCCTTGAAAATGT |
| T. tepi_3p_27975 |  | AATGTCGTCATGCCGCAAATGA |
| T. tepi_5p_23682 | AMRN01000006.1_23682 | TAAAAGAAGACGGGCACCTTAT |
| T. tepi_3p_23682 |  | GCCCGTCTTCTTTTGTTTTGTG |
| T. tepi_5p_25817 | AMRN01000007.1_25817 | CCGAACATCACAGCAAAACCCG |
| T. tepi_3p_25817 |  | CCTTGCGGGTTTTGTTTTGCCT |
| T. tepi_5p_33046 | AMRN01000010.1_33046 | AATCATCGATCCGTTGATCTTC |
| T. tepi_3p_33046 |  | AACTTTGTGCAGCTGTTTGGTC |
| T. tepi_5p_29936 | AMRN01000008.1_29936 | CGCGGCGGTGGCGTTGCCGAAC |
| T. tepi_3p_29936 |  | AACGTGATGGCGTCATGCACCG |
| T. tepi_5p_2196 | AMRN01000001.1_2196 | CATGACACCGACCCGGCATTGC |
| T. tepi_3p_2196 |  | TCATGCTGGGTCCAGTCGGCCT |
| T. tepi_5p_19433 | AMRN01000004.1_19433 | CGGTTGCAATTGCGACCACCAC |
| T. tepi_3p_19433 |  | CCTTATAGCGGAATGCGCCCTG |
| T. tepi_5p_6592 | AMRN01000001.1_6592 | ATCAAAAAGGCGGAGCTGATTT |
| T. tepi_3p_6592 |  | CTCCGCCTTTTTTTTGTTCGAG |
| T. tepi_5p_7069 | AMRN01000001.1_7069 | AAGGGCGGTAAACCGTTCTTTG |
| T. tepi_3p_7069 |  | CTTTGGCATCTGTGTTGGCATG |
| T. tepi_5p_3446 | AMRN01000001.1_3446 | TCAGTGCCGCGCACTTCGTCGT |
| T. tepi_3p_3446 |  | AGTGCGGCATCGACCTGATCAC |
| T. tepi_5p_36487 | AMRN01000013.1_36487 | GGGGGGAAAAGTTCCCTTGCCG |
| T. tepi_3p_36487 |  | TTGCCGAACGGCTGAAAGAGCT |
| T. tepi_5p_28590 | AMRN01000008.1_28590 | CATTCTGGCGCTATGCCGGTAT |
| T. tepi_3p_28590 |  | TATGCGTTTTGTCCATGACAAA |
| T. tepi_5p_19555 | AMRN01000004.1_19555 | CAATCTGTTGCAGTGCCTGATC |
| T. tepi_3p_19555 |  | CTGATCTGCTTCGTTACGGATA |
| T. tepi_5p_5471 | AMRN01000001.1_5471 | TGCCTATCGCGTCGACGAGGTG |
| T. tepi_3p_5471 |  | TGTCGAGGCGGCTGGTCTGCGT |
| T. tepi_5p_572 | AMRN01000001.1_572 | CACCGATGTCGAAAGATCTTCG |
| T. tepi_3p_572 |  | TTCCATATCGTGTCGTTATTCA |
| T. tepi_5p_20938 | AMRN01000005.1_20938 | AATTGTATGTGCAATAATGCGA |
| T. tepi_3p_20938 |  | GCGTTCGGAGGATTGCACATGC |
| T. tepi_5p_22942 | AMRN01000006.1_22942 | CTGCGACACAGTTGTCGCAGGC |
| T. tepi_3p_22942 |  | TGGGGTGGCTTTTCTTTTTCGG |
| T. tepi_5p_34995 | AMRN01000011.1_34995 | CAAATCCCTTGACCTGGGATCA |
| T. tepi_3p_34995 |  | ATTTTCCTAGCTTTTTCTCTCA |
| T. tepi_5p_5603 | AMRN01000001.1_5603 | CGCCTATTGGAATTCGGACGCG |
| T. tepi_3p_5603 |  | CGGACGCGATGCTGCTGCGTAT |
| T. tepi_5p_30879 | AMRN01000008.1_30879 | TTTCGACAATGACGATGTGTCG |
| T. tepi_3p_30879 |  | TGGCGTCGTTGCCGAAAGCTGG |
| T. tepi_5p_11782 | AMRN01000002.1_11782 | TGTGATGGTTTCTTCTATCGCA |
| T. tepi_3p_11782 |  | GTCGGTGGCGGTAACACCGCGG |
| T. tepi_5p_25251 | AMRN01000007.1_25251 | TTTCAACAACGCCCGTTGATTG |
| T. tepi_3p_25251 |  | ATTGAAATCCCCCGCCTAAACC |
| T. tepi_5p_27927 | AMRN01000007.1_27927 | ATTTTGTACCTGATGAAACGGC |
| T. tepi_3p_27927 |  | CGTTTTGTTAGGTGTTAACCTG |
| T. tepi_5p_17905 | AMRN01000004.1_17905 | AAGATGTCCGTAATTCGCGCGG |
| T. tepi_3p_17905 |  | CATCAACCTGTTGAACGGGTTG |
| T. tepi_5p_21204 | AMRN01000005.1_21204 | ATCGTCACCTTTGATGGTAACC |
| T. tepi_3p_21204 |  | TAACCTTTACGAAGCCATTGCC |
| T. tepi_5p_15757 | AMRN01000003.1_15757 | TATGCCAACAATCCGACCGGGT |
| T. tepi_3p_15757 |  | GCGGTCTGGATGTTGGCCTGCC |
| T. tepi_5p_26936 | AMRN01000007.1_26936 | AACAAAACCCGCAAGGCCAATG |
| T. tepi_3p_26936 |  | TTGCGGGTTTTGCTGTGATGTT |
| T. tepi_5p_29436 | AMRN01000008.1_29436 | AAAGGTGTATTTGTTGAAGACG |
| T. tepi_3p_29436 |  | TGCGTTCTTCAGCGCACCTTCA |
| T. tepi_5p_16459 | AMRN01000003.1_16459 | AACGGCGTTGGTTGAAACGGTT |
| T. tepi_3p_16459 |  | TTTCAGGATGAAAATGACCGCC |
| T. tepi_5p_18168 | AMRN01000004.1_18168 | AAAGGCATCTATATGCCGGTCG |
| T. tepi_3p_18168 |  | GGTCGGTCTCCTTGGCTTTCCT |
| T. tepi_5p_17794 | AMRN01000004.1_17794 | CTGAGCCTTGAGGCCTTTGGCG |
| T. tepi_3p_17794 |  | TTGGCGGTGATCTTCATGACTT |
| T. tepi_5p_13698 | AMRN01000003.1_13698 | AGCAAAAGCTGCCTAATTAAGG |
| T. tepi_3p_13698 |  | CTTCTGCTTTACAGACAGAATT |
| T. tepi_5p_21300 | AMRN01000005.1_21300 | TGCGCGACGATCATCATCACGG |
| T. tepi_3p_21300 |  | GATCATGATGATCATCGTCGTG |
| T. tepi_5p_31639 | AMRN01000009.1_31639 | CAGCGGCCTTGTTGATCCGCTT |
| T. tepi_3p_31639 |  | CGCTTATCTATTTGCCGTGATC |
| T. tepi_5p_17561 | AMRN01000004.1_17561 | AACTGGTCTCGGTTCTGACCTG |
| T. tepi_3p_17561 |  | ATGTCGAAACCGAAACCATGCC |
| T. tepi_5p_33271 | AMRN01000010.1_33271 | TGTCTTTTTCTGACGTTTTTTC |
| T. tepi_3p_33271 |  | CGTTTTTTCTCAAAAAAGGGTT |
| T. tepi_5p_11264 | AMRN01000002.1_11264 | TTGTCTGTCAAACAGGCAAGGA |
| T. tepi_3p_11264 |  | AAGGATTGCGGTCGGCCTTACT |
| T. tepi_5p_8741 | AMRN01000002.1_8741 | TGCAGTTCGATGAAGTTCTTCT |
| T. tepi_3p_8741 |  | CCGACCTTTTCCAAGGATGACA |
| T. tepi_5p_34376 | AMRN01000011.1_34376 | TGACGCAGAGGCTTTCTCTCAT |
| T. tepi_3p_34376 |  | AGGTGGCCTTTGGATCACCCGG |
| T. tepi_5p_33867 | AMRN01000010.1_33867 | GTCCGACTGGCTTGGATAAATC |
| T. tepi_3p_33867 |  | AGCTGTAATCACTGCGCACCAT |
| T. tepi_5p_29208 | AMRN01000008.1_29208 | CCGGTGCTTCTGTGTGCCGACG |
| T. tepi_3p_29208 |  | ATGGCTGACATTCCGCGTCTGG |
| T. tepi_5p_19094 | AMRN01000004.1_19094 | TTCAAGGATTATCTTGCCACAC |
| T. tepi_3p_19094 |  | TTTGGCGAGATGGGCCTTGCCG |
| T. tepi_5p_8405 | AMRN01000002.1_8405 | GTCGGCGTTGTCGCGCTGTTCA |
| T. tepi_3p_8405 |  | TTCAAGGAGCCGCTGCATGTTG |
| T. tepi_5p_8120 | AMRN01000001.1_8120 | TGCGCCGTTCGGCCTATGTCGC |
| T. tepi_3p_8120 |  | CACCGTTTTGATGCCGTCCTTT |
| T. tepi_5p_33254 | AMRN01000010.1_33254 | TGCTGGAACAGCATTTCGGTGT |
| T. tepi_3p_33254 |  | TTCGGTGTTTGCGACTTCAAAG |
| T. tepi_5p_18865 | AMRN01000004.1_18865 | TGTCGGGCGGGCGCGGCGGTGT |
| T. tepi_3p_18865 |  | CAATGAGCCCTATATCGCGGTG |
| T. tepi_5p_2711 | AMRN01000001.1_2711 | CCGGATATAACTATCCGTCGGG |
| T. tepi_3p_2711 |  | TCGGGCAGCACTTGTTGGCCGG |
| T. tepi_5p_5680 | AMRN01000001.1_5680 | AGGGTGCAGAAGCATTCGTTCG |
| T. tepi_3p_5680 |  | GTTCGCCTTTTTTATAAGGATG |
| ***T. xiamenensis* M-5^T^** | | |
| T. xiam_5p_26826 | CP004388.1_26826 | AGACGTGACCTTCGGGTCGCGT |
| T. xiam_3p_26826 |  | CGTCTTTTTTATTGTCTGGTGG |
| T. xiam_5p_11702 | CP004388.1_11702 | CAATTAAAAACCCCCTCAGGCG |
| T. xiam_3p_11702 |  | AGGGGTTTTTTAATTGGTAGCC |
| T. xiam_5p_37696 | CP004388.1_37696 | TCATTTTTGCGCCGACCCTATT |
| T. xiam_3p_37696 |  | CGACCCTATTCATTTCTGAATT |
| T. xiam_5p_39114 | CP004389.1_39114 | GATATCAGACCTTACCTGCACC |
| T. xiam_3p_39114 |  | CGGTGCAGGTAAGGTCTGTAGG |
| T. xiam_5p_27030 | CP004388.1_27030 | CGCGGTGAGATTTTCGCCGCGT |
| T. xiam_3p_27030 |  | CGCGTTTTTTGTCGGTACCGCA |
| T. xiam_5p_6808 | CP004388.1_6808 | GCGCGGGAATGACGGTTTTTGG |
| T. xiam_3p_6808 |  | TCACTCCCGCGAAGGCGGGAGT |
| T. xiam_5p_32390 | CP004388.1_32390 | CGGTTGCATTGGCAAAGGCATC |
| T. xiam_3p_32390 |  | AGGCATCGACGGATGCCGGTGC |
| T. xiam_5p_34788 | CP004388.1_34788 | TTATACATTGAGAGGCAGAAGG |
| T. xiam_3p_34788 |  | CTTCTGCCTCTTTTTCGTTTGA |
| T. xiam_5p_8112 | CP004388.1_8112 | TGCCAGCCATCGAAAACCGATT |
| T. xiam_3p_8112 |  | GCCGGTTTTCTGTGGCTGATTT |
| T. xiam_5p_12450 | CP004388.1_12450 | ATCCGTCAGCGCGTTCTGGCTG |
| T. xiam_3p_12450 |  | CTGGATTATTCGCAACGCTGCT |
| T. xiam_5p_17371 | CP004388.1_17371 | CTTTTCCATCTGTTTGGCGGCA |
| T. xiam_3p_17371 |  | TTATTCAGATCTGGAAAAGCTG |
| T. xiam_5p_6543 | CP004388.1_6543 | TATAAAAGGCCCGCCGTTCTGG |
| T. xiam_3p_6543 |  | AGAATTGCGGGTCTTTTTTGGT |
| T. xiam_5p_18736 | CP004388.1_18736 | ATCGCCCGCCATTCGGGTGCTT |
| T. xiam_3p_18736 |  | TCCGAAATGCGTTTGTCGCGAA |
| T. xiam_5p_38869 | CP004388.1_38869 | AGATTCGTTTTTTATCTATAAT |
| T. xiam_3p_38869 |  | TATCTATAATTTTGAATGCGAT |
| T. xiam_5p_35854 | CP004388.1_35854 | AGTGCGGATCGCGCCTCGATGC |
| T. xiam_3p_35854 |  | CGGGGCCGGATCCGTTGACGAG |
| T. xiam_5p_36112 | CP004388.1_36112 | ACGAAACGAAAGACCCCCATTA |
| T. xiam_3p_36112 |  | TGGGGGTCTTTGCGTTTCAGGT |

*: The identified putative miRNAs were named according to <name of bacteria>_<location of miRNA in the precursor>_<position of miRNA in the genome>. All sequences have 22 nt in length.
